# Research on energy optimization control strategy for parallel hybrid tractor based on AIPSO

**DOI:** 10.1371/journal.pone.0315369

**Published:** 2025-02-13

**Authors:** Xiaohui Liu, Yiwei Wu, Jingyun Zhang, Yifan Zhao, Yangming Hu, Xianghai Yan

**Affiliations:** 1 College of Vehicle and Traffic Engineering, Henan University of Science and Technology, Luoyang, Henan, China; 2 State Key Laboratory of Intelligent Agricultural Power Equipment, Luoyang, Henan, China; Vellore Institute of Technology, INDIA

## Abstract

In the research on energy optimization control for parallel hybrid tractors, torque has been identified as a crucial factor influencing the tractor’s fuel economy, operational efficiency, and agricultural development. This study focuses on the tractor’s overall demand torque as the primary research parameter and designs a comprehensive scheme for the parallel hybrid tractor. The study includes the design of power system parameters and the construction of a dynamic model for the entire machine. Based on this, an energy optimization control strategy, termed adaptive immune particle swarm optimization fuzzy control strategy (AIPSOFCS), is proposed. Simulation analysis is performed using representative plowing conditions, and AIPSOFCS is compared with the power follow control strategy (PFCS) and fuzzy control strategy (FCS). The results indicate that AIPSOFCS demonstrates higher fuel economy and operational efficiency compared to PFCS and FCS. In the plowing conditions, the fuel economy of AIPSOFCS is reduced by 8.45% and 2.93% compared to PFCS and FCS, respectively. In the rotary tillage conditions, the fuel economy of AIPSOFCS is reduced by 2.40% and 4.07% compared to PFCS and FCS, respectively. Finally, hardware-in-the-loop (HIL) testing of the controller confirms the effectiveness of AIPSOFCS. This research is of significant importance for enhancing the fuel economy and operational efficiency of parallel hybrid tractors and provides theoretical support and reference for the future.

## Introduction

The new agricultural business models and production modes have raised higher demands for ecology, energy saving, and environmental protection. Tractors, as representative agricultural machinery, are the primary power source for agricultural production. Traditional fuel-powered tractors can no longer meet the demands of agricultural development. However, pure electric tractors are still limited by battery technology in terms of range and cannot yet be widely promoted in the market [1, 2]. The diesel-electric hybrid tractor combines the advantages of both pure electric tractors and traditional fuel-powered tractors. It has become an effective solution to the current challenges in the agricultural machinery industry and is also a key research direction for the development of agricultural equipment towards ecology, energy saving, and environmental protection [3, 4]. In a parallel hybrid tractor, power can be supplied either by the power source alone or jointly by the motor and engine. Compared to series hybrid tractors, parallel hybrid tractors do not require secondary energy conversion, leading to higher energy utilization. In contrast to series-parallel hybrid tractors, parallel hybrid tractors have a simpler structure, making development and maintenance less complex. Current research on parallel hybrid tractors primarily focuses on power transmission system parameter matching, control strategy development and optimization, and tractor testing and validation. Among these, the energy optimization control strategy is the core and key to the overall control of the tractor. Therefore, designing a reasonable energy optimization control strategy for parallel hybrid tractors is an effective way to improve overall fuel economy and operational efficiency, and it is of significant importance for the development of agricultural machinery toward ecological, energy saving, and environmental protection goals [5, 6].

Energy optimization control strategies can be mainly classified into two types: optimization-based and rule-based control strategies. Rule-based energy optimization control strategies are widely used in practical engineering applications. They are relatively simple in design, easy to interpret, and highly reliable, meeting the needs of engineering applications. Zhang Zhen et al. [7] applied long short-term memory (LSTM) networks to the proximal policy optimization (PPO) algorithm, developing a PPOLSTM energy management strategy for plug-in hybrid electric vehicles (PHEVs) to achieve optimal operating mode switching. The energy consumption rates improved by 18.51% and 15.74% under the WLTC and NEDC cycles, respectively. Liu Kai [8] used the PFCS combined with the Grey Wolf Optimization algorithm to optimize the power threshold parameters for the range extender’s start-stop function, effectively reducing engine start-stop frequency and improving overall vehicle fuel economy by 6.89%. Chen Zhixi et al. [9] proposed a control strategy that adjusts the power output mode based on changes in load-following thresholds, battery State of Charge (SOC), and engine speed, which effectively improved overall vehicle fuel economy. Wang Yong et al. [10] designed dual fuzzy control strategies and a dual fuzzy control strategy optimized by particle swarm optimization (PSO), which protected the power battery and significantly enhanced vehicle performance. Wang Chunguang [11] designed a fuzzy rule-based control strategy for parallel hybrid tractors under transportation conditions and conducted joint simulations in ADVISOR and Simulink. The results showed that the tractor’s fuel economy in transportation mode was superior to that of electric assist strategies. The research indicates that the performance of energy optimization control strategies based on rules is highly dependent on the rationality of the control rules, and ensuring the comprehensive performance of the tractor can be challenging.

Optimization-based energy optimization control strategies are mainly divided into instantaneous optimization and global optimization, based on optimization theories and methods. These strategies are designed according to different objective functions and constraints. Fengqi Zhang et al. [12] considering the aging characteristics of power batteries, conducted an in-depth analysis of the impact of three energy optimization control strategies on vehicle performance: dynamic programming (DP), pontryagin’s minimum principle (PMP), and equivalent consumption minimization strategy (ECMS). Du Aimin et al. [13] used the particle swarm optimization (PSO) algorithm to optimize key parameters of the ECMS, resulting in a significant reduction in vehicle fuel consumption. Stefano Radrizzani et al. [14] proposed an ECMS that addresses built-in speed-tracking requirements. This control strategy was evaluated under real tractor operating conditions and achieved an average fuel saving of 14%. Zhu Zhen [15] designed a parallel hybrid tractor power system using HMCVT and proposed a fuzzy adaptive equivalent consumption minimization strategy (FA-ECMS). This strategy incorporates a fuzzy PI controller to dynamically adjust the equivalent factor, reducing the tractor’s overall fuel consumption by 6.71%. Li Yanying et al. [16] developed an integrated electromechanical-hydraulic power bond graph model covering the energy system, drive system, and lifting system of agricultural tractors. They proposed a hierarchical dynamic programming energy management strategy, which significantly reduced equivalent hydrogen consumption in both plowing and transportation conditions. Zhang Junjiang et al. [17] proposed an instantaneous optimization-based energy-saving control strategy using motor torque and diesel engine torque as control variables, and battery state of charge (SOC) as a state variable. This strategy reduced equivalent fuel consumption by 4.7% and 6.31% in rotary tillage and plowing conditions, respectively, compared to power-following control strategies. Research indicates that instantaneous optimization strategies can achieve optimal fuel economy in the short term or within a few seconds, but cannot ensure global optimality across all operating conditions. Global optimization strategies can achieve overall optimal performance based on all vehicle operating conditions, but the complexity of the tractor’s operating environment makes it challenging to use complete operational information as the control premise.

As noted, current research on energy optimization control strategies primarily focuses on automobiles, with relatively little attention given to parallel hybrid tractor energy optimization control. Early research on energy optimization control for parallel hybrid tractors mostly concentrated on tractor speed as the dominant factor. While this approach effectively controls tractor speed, it does not adequately address the tractor’s overall torque requirements. In practical agricultural operations, meeting the overall torque demand is a crucial factor for tractor performance. Therefore, this study focuses on the tractor’s overall torque demand as the main research parameter. Building on the PFCS, the study employs an FCS with the SOC of the power battery and the overall torque demand as fuzzy control input variables, and engine torque as the fuzzy control output variable. Based on this, an AIPSOFCS is proposed to reduce the tractor’s equivalent fuel consumption and enhance fuel economy.

The main research content of this paper has been as follows: Chapter 1 has analyzed the topology and working characteristics of parallel hybrid tractors, using plowing conditions as the basis for force analysis, and has completed the design of the power system structure and parameters. Chapter 2 has used forward modeling to establish models of the main components of the parallel hybrid tractor, including the driver model, motor model, and power battery model, and has built an overall simulation model. Chapter 3 has proposed an energy optimization control strategy based on adaptive immune particle swarm optimization (AIPSO) for fuzzy control membership functions, building on traditional power-following control strategies and fuzzy control strategies. This strategy has aimed to better meet the tractor’s torque requirements and improve fuel economy and operational efficiency. Chapter 4 has focused on the tractor’s plowing conditions and rotary tillage conditions, performing a simulation analysis of the AIPSOFCS fuel economy based on the overall dynamic model in the Matlab/Simulink environment, and has compared it with PFCS and FCS. A HIL platform has been constructed to validate the effectiveness and feasibility of the three energy optimization control strategies. Future research directions have also been discussed. Chapter 5 has summarized the research content and experimental results of this paper.

### Overall scheme of the parallel hybrid tractor power system

The parallel hybrid tractor achieves a parallel connection between the engine and motor through an electromechanical coupling device, operating as two independent drive systems. These systems can drive the tractor separately or together. The power system is the core component of the parallel hybrid tractor and is a key distinguishing feature from other tractors. A well-designed power system is crucial for ensuring the efficient operation of the parallel hybrid tractor, as it is responsible for coordinating the work of the motor and engine and optimizing the use and distribution of energy [18]. This chapter has completed the design of the power system structure and parameters, using plowing conditions as the basis for force analysis.

### Power system structure of parallel hybrid tractor

**Fig 1:**
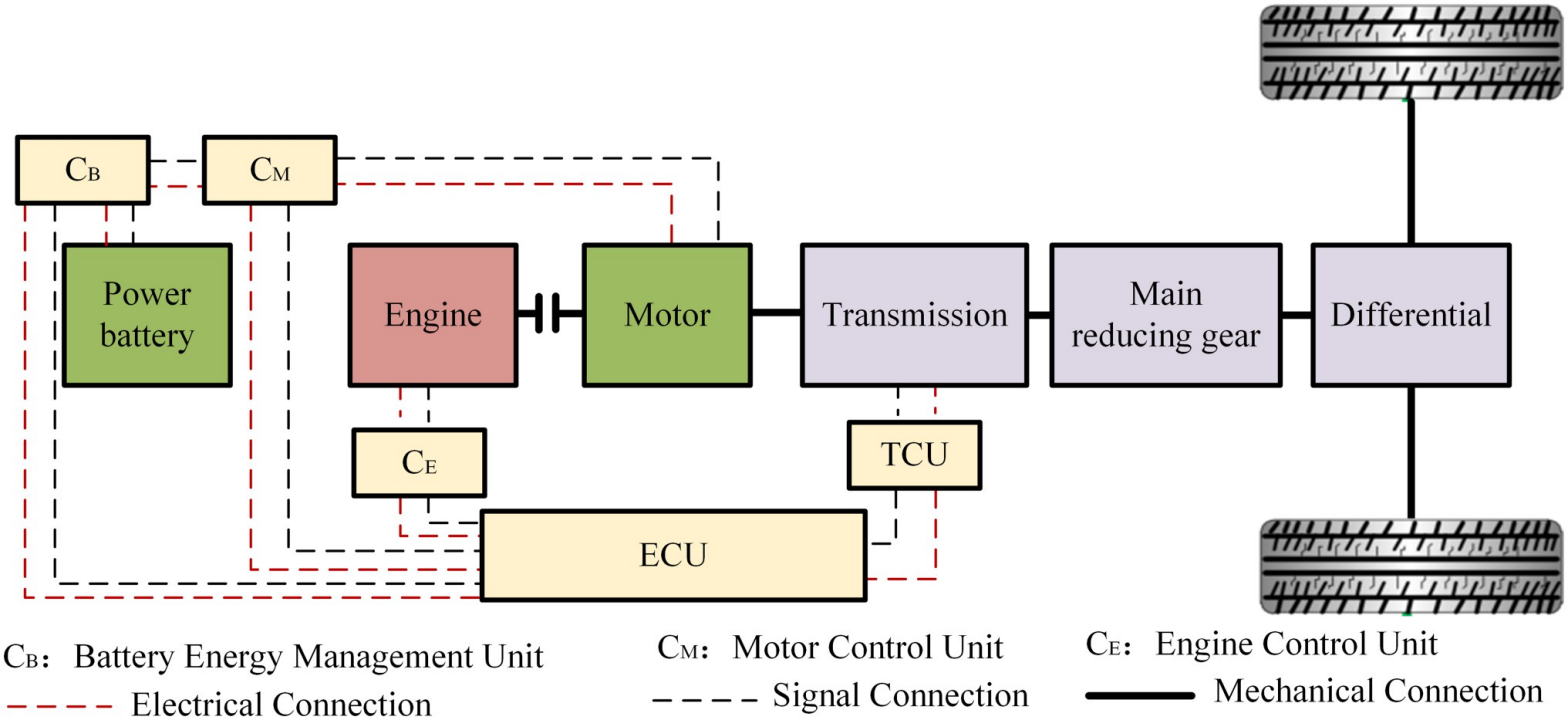
Schematic diagram of the power system of the parallel hybrid tractor.

The overall structure of the parallel hybrid tractor power system studied in this paper is shown in Fig 1. Both the engine and motor of the tractor can provide power directly, with different power combinations capable of meeting the power requirements for various agricultural operations. The power system of this parallel hybrid tractor consists of a power battery, diesel engine, motor, transmission, main reduction gear, differential, and related control system components. The transmission, main reduction gear, and differential constitute the drivetrain of the power system.

**Fig 2:**
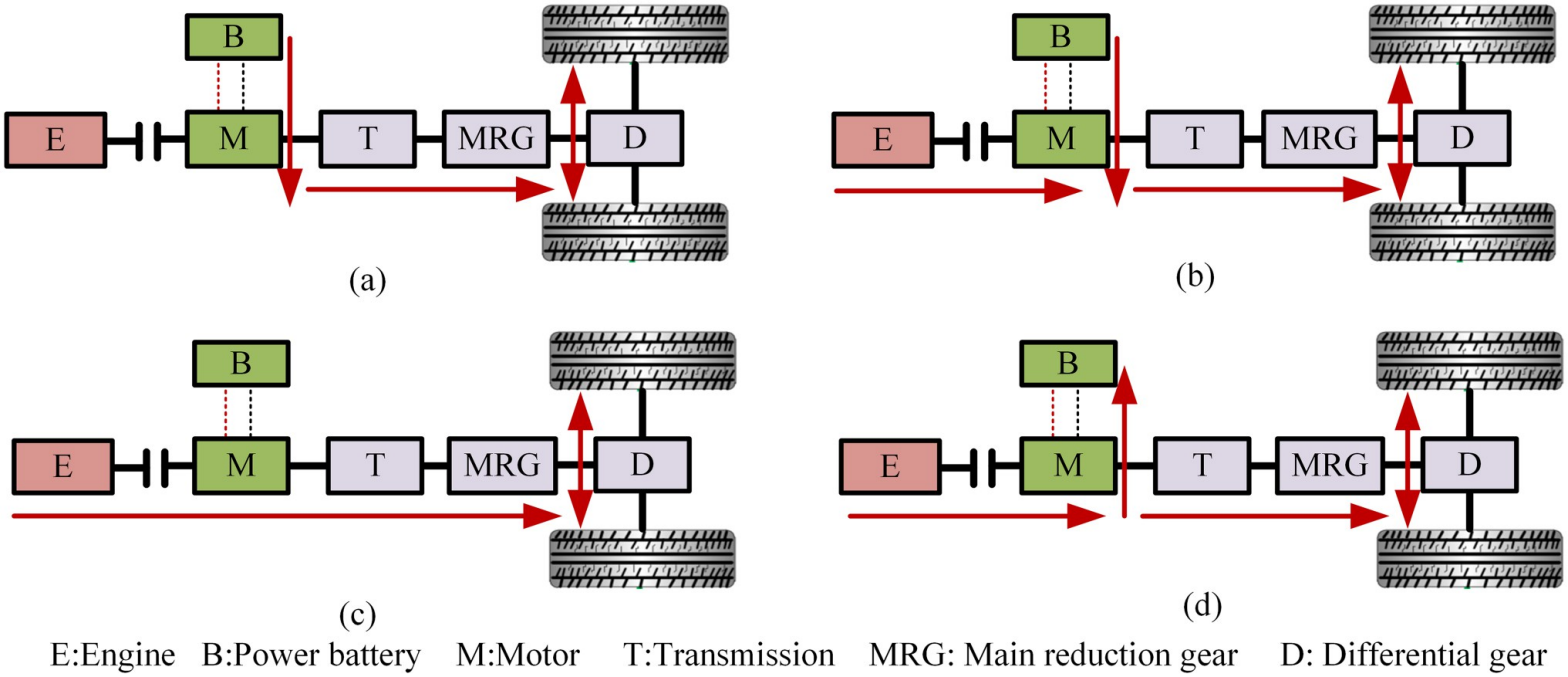
Working modes of the hybrid system. (a) Pure electric driving mode. (b) Hybrid driving mode. (c) Pure engine driving mode. (d) Driving with charging mode.

The parallel hybrid tractor with this configuration can achieve the following four operational modes, as illustrated in Fig 2:

1. Pure electric driving mode: This mode is suitable for situations where the tractor is starting or operating under low load conditions, and the electric motor can efficiently meet the power requirements of the entire system. In this mode, the internal combustion engine is shut off, and the power battery supplies electrical energy to the motor. The motor then transmits power to the drive wheels through the transmission system to drive the tractor. The power transmission route is depicted in Fig 2(a).
2. Hybrid driving mode: This mode is suitable for high-load conditions, such as tilling or plowing operations. In this mode, both the engine and the electric motor provide power, which is then combined through the transmission shaft and delivered to the drive wheels to operate the tractor. The power transmission route is illustrated in Fig 2(b).
3. Pure engine driving mode: This mode is appropriate for medium to low-load operations or the SOC of the power battery is low and the engine can efficiently meet the power demands of the entire system. In this mode, the motor is not active, and the engine transmits power through the transmission system to the drive wheels to operate the tractor. The power transmission route is shown in Fig 2(c).
4. Driving with charging mode: This mode is applicable when the engine is operating independently and the SOC of the power battery is low. The engine drives the tractor for normal operation, while the remaining power is used by the motor to charge the power battery. The power transmission route is depicted in Fig 2(d).

### Design of power system parameters for parallel hybrid tractors

The rationality of parameter design for each component in the tractor power system directly impacts key performance metrics, including overall comfort, power performance, fuel economy, and emissions. This study selects the plowing operation, a typical working condition for tractors, as the basis for dynamic analysis to determine the rated traction force of the entire machine and conduct the parameter design.

#### Overall power requirements.

During low-speed operations of the tractor, air resistance and acceleration resistance can be neglected. Under plowing conditions, the parallel hybrid tractor must overcome both the plowing resistance and other resistances along the direction of travel. With a travel speed of approximately 4–10 km/h, the relationship between the tractor’s driving force *F*_*t*_ and other resistances is given by:

$$F_{t}=F_{p}+F_{f}+F_{i}$$ (1)

where *F*_*t*_ represents the driving force, N; *F*_*p*_ represents the plowing force, N; *F*_*f*_ represents the rolling resistance, N; *F*_*i*_ represents the slope resistance under plowing conditions, N.

In which the magnitude of the plowing resistance along the direction of travel is related to the parameters of the moldboard plow and the plowing depth:

$$F_{p}=zbhk$$ (2)

where *z* represents the number of moldboard plows; *b* represents the working width of a single moldboard plow, cm; *h* represents the plowing depth, cm; *k* represents the soil resistance, N/cm^2^.

The rated traction force of the tractor is typically related to the average traction resistance (plowing resistance) of the main matched agricultural implements under standard plowing conditions. Given the complexity and variability of operational conditions during tractor use, a power reserve of 10% to 20% must be allocated. The rated traction force *f*_*t*max_ and the maximum plowing resistance *f*_*p*max_ for the hybrid tractor are:

$$F_{t\text{max}}=(1.1∼1.2)F_{p}$$ (3)

$$F_{p\text{max}}=1.2F_{p}$$ (4)

Total power of the power system *P*_*Z*_:

$$P_{Z}=\frac{F_{t\text{max}}V_{1}}{3600\eta_{t}}$$ (5)

where *P*_*Z*_ represents the rated power of the power system, kW; *V*_1_ represents the maximum travel speed of the tractor during plowing operations, km/h; and *η*_*t*_ represents the driving efficiency of the tractor.

#### Parameter design of each component in the power system

During plowing operations, the tractor experiences variable loads and harsh conditions due to the agricultural environment. However, plowing conditions account for approximately 75% of the tractor’s entire operational lifecycle [19]. Therefore, this study selects plowing conditions as the primary research condition. Based on the drive system design methods outlined in the literature, the parameters for each component of the tractor are determined using a specific model tractor as a reference, as shown in Table 1.

**Table 1:** Main technical parameters of power system components of parallel hybrid power tractor.

Project	Parameters	Unit	Value
Tractor	Operating weight	kg	3600
	Wheel radius	m	0.9
Diesel engine	Rated power	kW	85
	Rated speed	r/min	2300
	Maximum torque speed	r/min	1500~1700
Motor	Rated power	kW	100
	Rated speed	r/min	2500
	Rated torque	N·m	360
Power battery	Capacity	A·h	110
	Rated voltage	V	540
Transmission	Gear ratio I	-	4.08
	Gear ratio II	-	3.24
	Gear ratio III	-	2.59
	Gear ratio IV	-	2.07
Main reduction gear	Transmission ratio	-	6.4
Differential	Transmission ratio	-	4.55

### Modeling of the power system for parallel hybrid tractors

To further investigate the impact of energy optimization control strategies on the fuel economy of tractors, precise power system modeling is essential. Therefore, based on the completion of the structural and parameter design of the parallel hybrid tractor’s power system, a forward modeling approach was used to establish models for its main components, including the driver model, the motor model, and the power battery model. Finally, a complete machine simulation model was developed.

### Driver model

The driver model is a subsystem primarily based on PI control. It uses the deviation between the desired velocity and the actual velocity as input, and the positions of the accelerator and brake pedal as output, to achieve follow-up control of the desired velocity under plowing conditions [20].

The input signals to the controller are:

$$v_{c}(t)=v_{req}(t)-v_{act}(t)$$ (6)

Where *v*_*c*_(*t*) represents the deviation between the desired velocity and the actual velocity at time *t*, km/h; *v*_*req*_(*t*) represents the desired velocity at time *t*, km/h; *v*_*act*_(*t*) represents the actual velocity at time *t*, km/h.

The input to the PI controller is:

$$u(t)=k_{p}v_{c}(t)+k_{i}\int v_{c}(t)dt$$ (7)

Where *u*(*t*) represents the pedal position signal, where a positive value indicates tractor acceleration and a negative value indicates tractor braking; *k*_*p*_ represents the proportional coefficient; *k*_*i*_ represents the integral coefficient.

### Motor model

**Fig 3:**
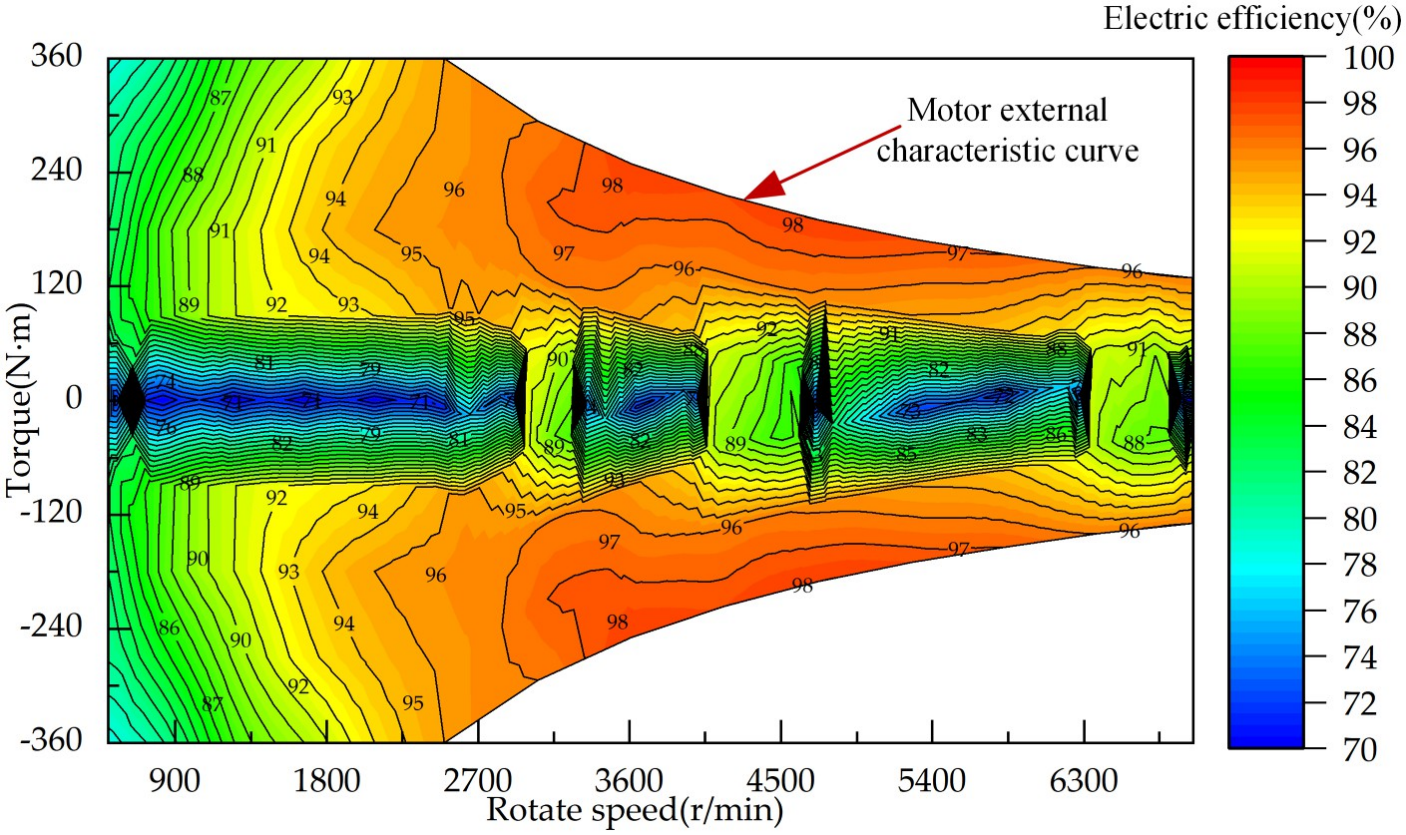
Diagram of motor efficiency.

This study considers only the efficiency of the motor’s external output and does not analyze the motor’s working principle; therefore, static modeling based on experimental data is utilized. Using the motor speed, torque, and efficiency data collected from motor tests, a motor efficiency map can be obtained, as shown in Fig 3. In this map, the linear part of the external characteristic curve represents the constant torque region of the motor, the hyperbolic part represents the constant power region, and the annular part corresponds to the equal-efficiency curves of the motor. The motor efficiency under different speed and torque conditions can be obtained from the efficiency map.

To select a high-performance permanent magnet synchronous motor for the tractor power drive system, the relationship among power, speed, and torque is expressed as follows:

$$P_{m}=\left\{\begin{array}{c} \frac{n_{m}T_{m}}{9550\eta_{m}},T_{m}>0\\ \frac{n_{m}T_{m}\eta_{m}}{9550},T_{m}\leq 0 \end{array}\right.$$ (8)

where *P*_*m*_ represents the motor power, kW; *n*_*m*_ represents the motor speed, r/min; *T*_*m*_ represents the motor torque, N·m; *η*_*m*_ represents the motor efficiency. If the motor power *P*_*m*_ is positive, the motor operates as a motor; if the motor power is negative, the motor functions as a generator.

**Fig 4:**
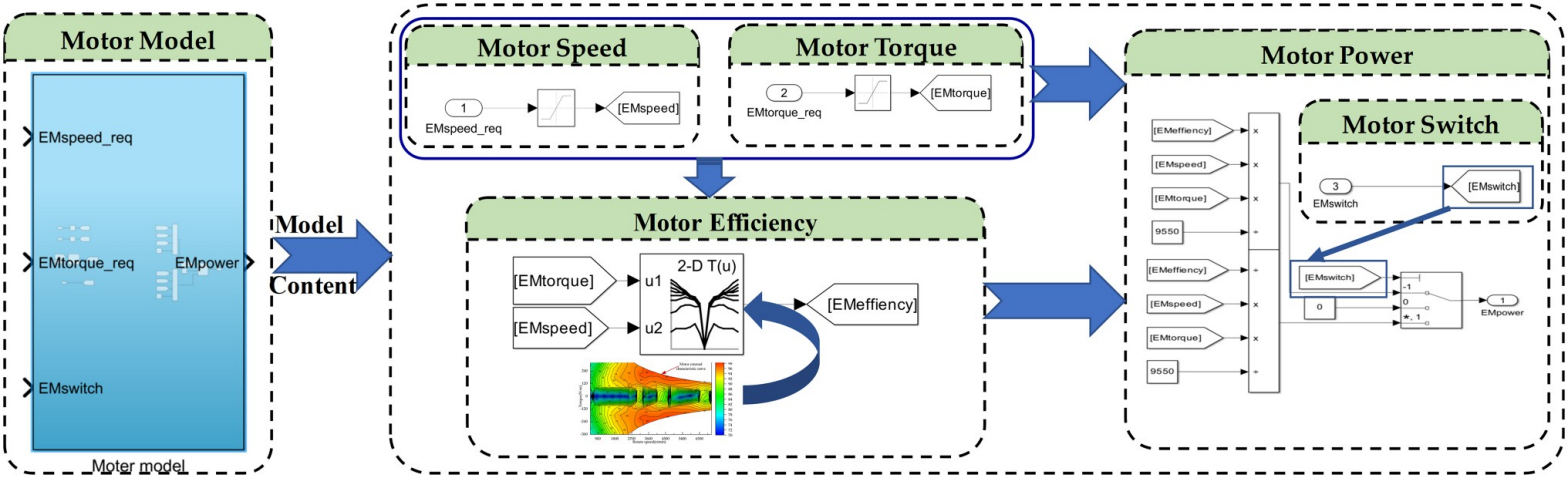
Motor Simulink model diagram.

The Simulink model of the motor is established as shown in Fig 4. In this model, the motor input speed is calculated from the tractor speed through the transmission system’s gear ratio. The motor input torque and the motor switch are allocated based on the control strategy. The motor efficiency is obtained from the efficiency map, and the final motor power is calculated using Eq (5).

### Power battery model

**Fig 5:**
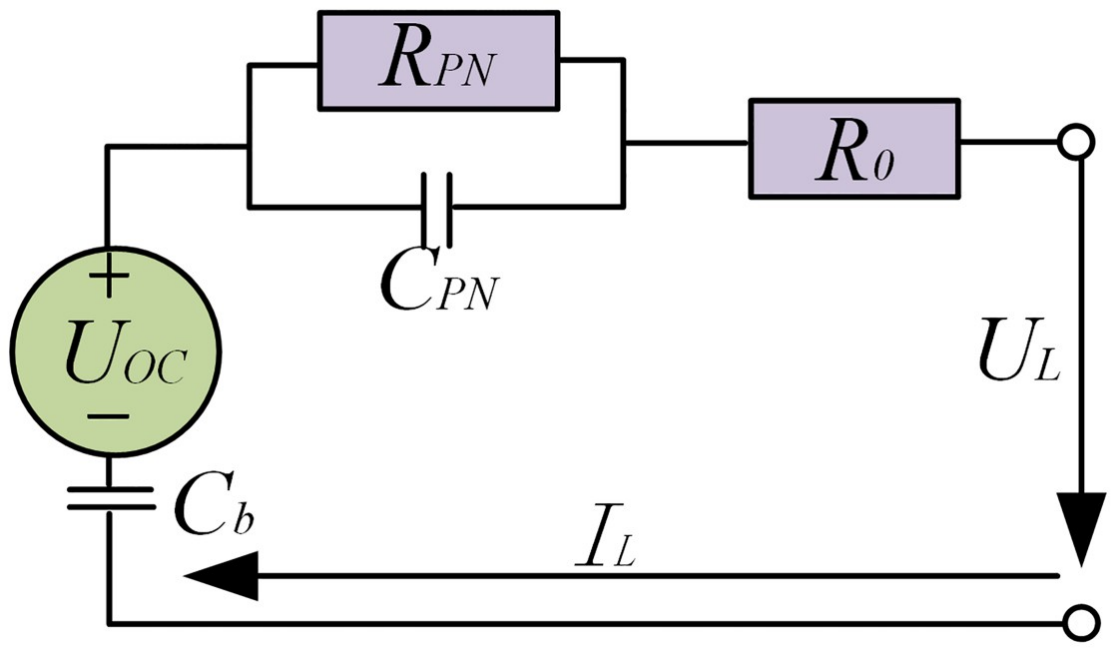
Equivalent circuit of power battery.

The power battery is a critical energy-balancing component in hybrid tractors, and the accuracy of its model significantly impacts the overall performance and efficiency of the system. Commonly used power battery models include the Rint model, Thevenin model, second-order RC model, and PNGV model. Among these, the PNGV model can more accurately account for variations in battery characteristics. Therefore, this study selects lithium iron phosphate (LiFePO_4_) batteries and employs the PNGV model to establish the physical battery model [21]. The equivalent circuit is shown in Fig 5.

From Kirchhoff’s voltage law and Kirchhoff’s current law, the relationship between the output voltage and input current in the PNGV model can be expressed as follows:

$$U_{OC}=R_{PN}I_{PN}+R_{0}I_{L}+U_{L}+\frac{1}{C_{b}}\left(\int I_{L}dt\right)$$ (9)

Where *U*_*OC*_ represents the open-circuit voltage (OCV), V; *R*_*PN*_ denotes the polarization resistance, Ω; *I*_*PN*_ is the polarization resistance current, A; *C*_*PN*_ refers to the polarization capacitance, F; *R*_0_ stands for the ohmic resistance, Ω; *U*_*L*_ is the load voltage, V; *I*_*L*_ denotes the circuit current, A; and *C*_*b*_ indicates the variation in OCV, F.

As a crucial decision factor in energy management, SOC plays a significant role in tractor energy management, enhancing battery capacity and energy utilization efficiency, preventing overcharging and deep discharging, and ensuring the safety of battery usage. SOC represents the ratio of the current battery charge to the total battery capacity:

$$SOC(t)=\frac{Q(t)}{Q_{sum}}$$ (10)

where *Q*(*t*) denotes the current capacity of the power battery, A·h; *Q*_*sum*_ represents the total capacity of the power battery, A·h.

The dynamic change in the SOC of the power battery is given by:

$$SOC(t)=\left\{\begin{array}{c} \frac{I_{batt}(t)\eta_{batt}}{Q_{sum}},I\left(t\right)<0\\ \frac{I_{batt}(t)}{Q_{sum}\eta_{batt}},I\left(t\right)>0 \end{array}\right.$$ (11)

Where *I*_*batt*_(*t*) < 0 represents the discharge process, *I*_*batt*_(*t*) > 0 denotes the charging process; *η*_*batt*_ is the coulomb efficiency of the power battery, which indicates the ratio of the discharged amount to the charged amount over a cycle.

**Fig 6:**
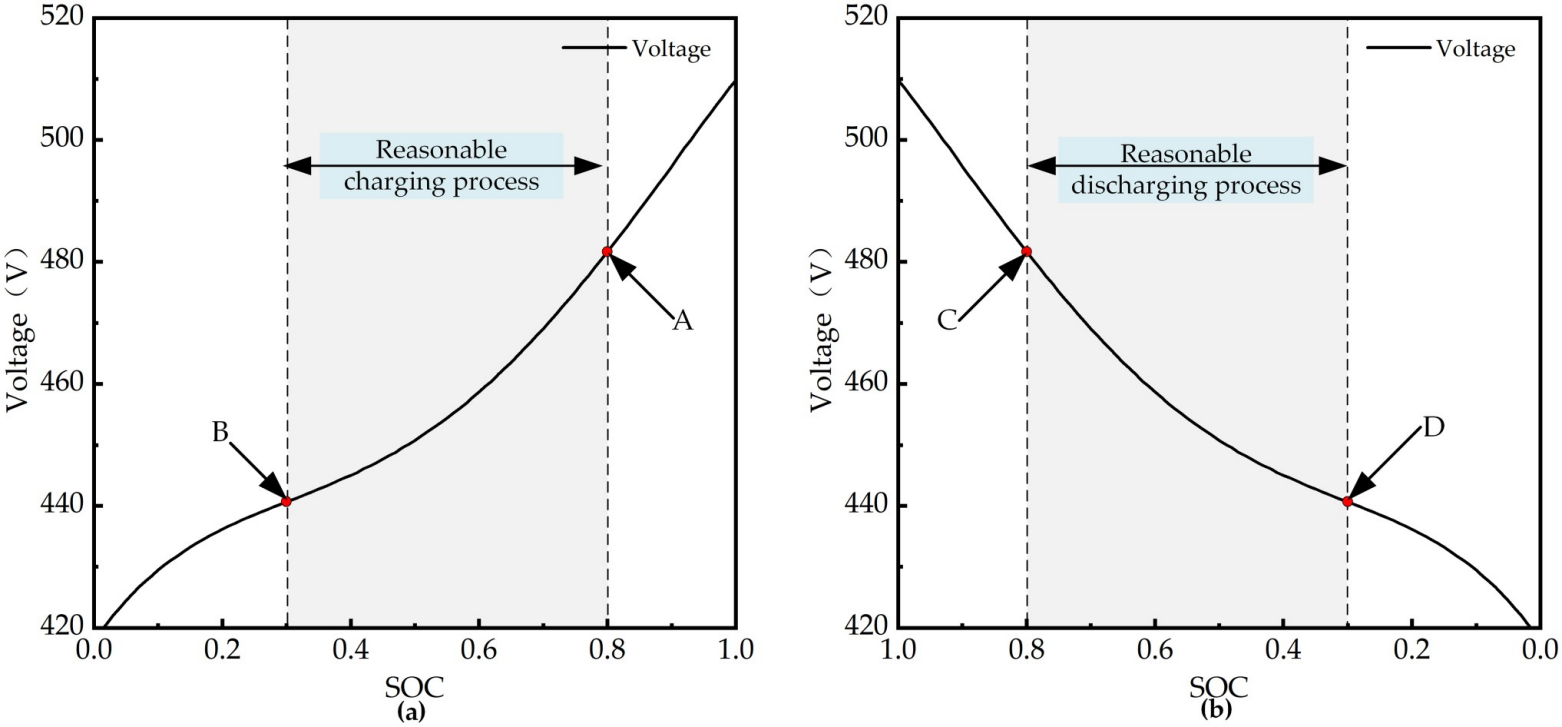
Relationship between SOC and OCV during charge and discharge processes. (a) Charge cycle: The OCV increases with SOC. (b) Discharge cycle: The OCV decreases with SOC.

During the charging process, the relationship between SOC and open OCV is shown in Fig 6(a); during the discharge process, the relationship is depicted in Fig 6(b). The tractor operates under the "shallow charge and shallow discharge" principle. In this study, the SOC range is between 0.3 and 0.8. Points A and C represent the highest SOC levels during operation, while points B and D indicate the lowest SOC levels.

**Fig 7:**
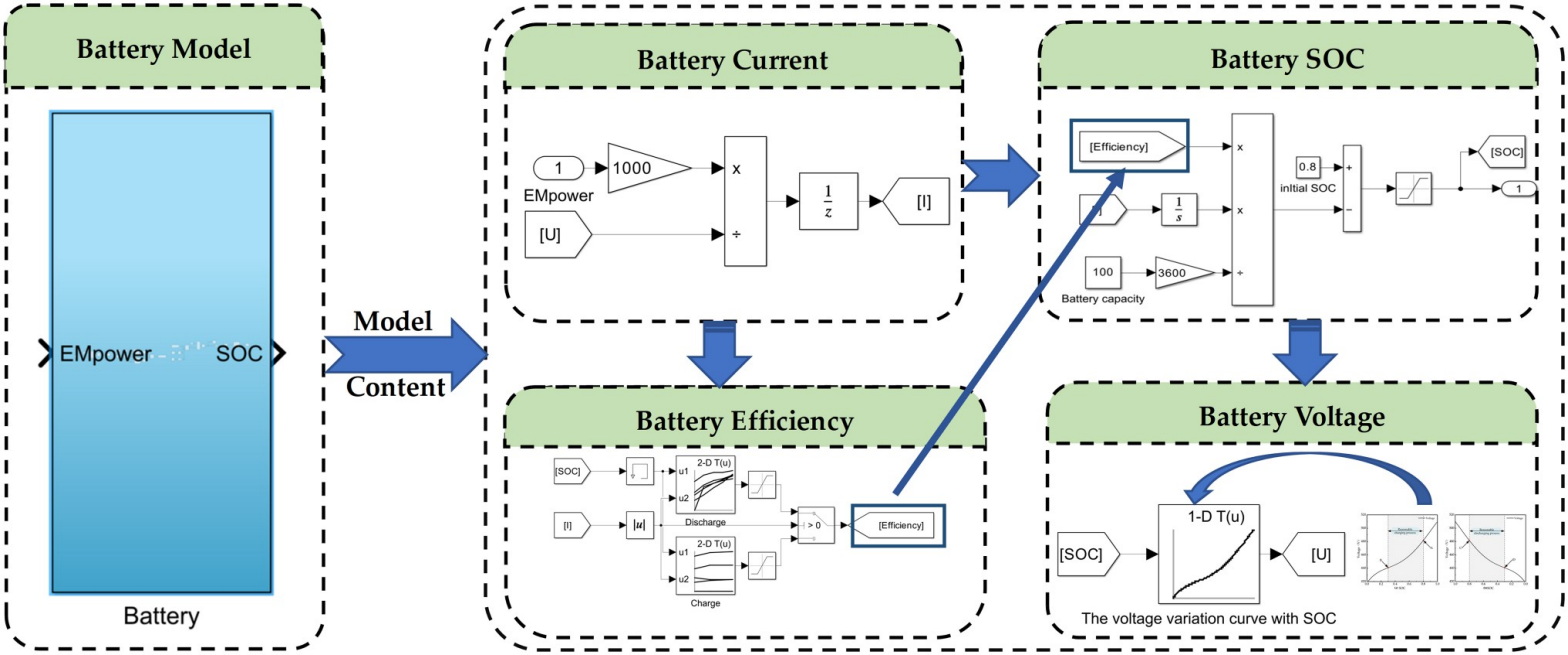
Battery Simulink model diagram.

Following the reverse simulation design approach, the input to the power battery model is the battery power. From this input, the battery’s current, voltage, and SOC can be calculated. Therefore, a Simulink model of the power battery is established as shown in Fig 7. The battery charging and discharging efficiencies are determined from a lookup table. Based on the power and voltage, the battery current is calculated, and subsequently, the SOC of the power battery is determined. The battery voltage can be obtained from the lookup table according to Fig 6.

### Parallel hybrid tractor energy optimization control strategy

The rationality of the energy optimization control strategy for a hybrid tractor directly affects the overall performance of the machine. For a parallel hybrid tractor, three different control strategies are proposed to effectively manage the magnitude and direction of energy flow between the motor and the engine. These strategies are PFCS, FCS, and AIPSOFCS.

### Traditional control strategies

The traditional control strategies studied in this paper primarily include the PFCS and the FCS.

#### PFCS

**Fig 8:**
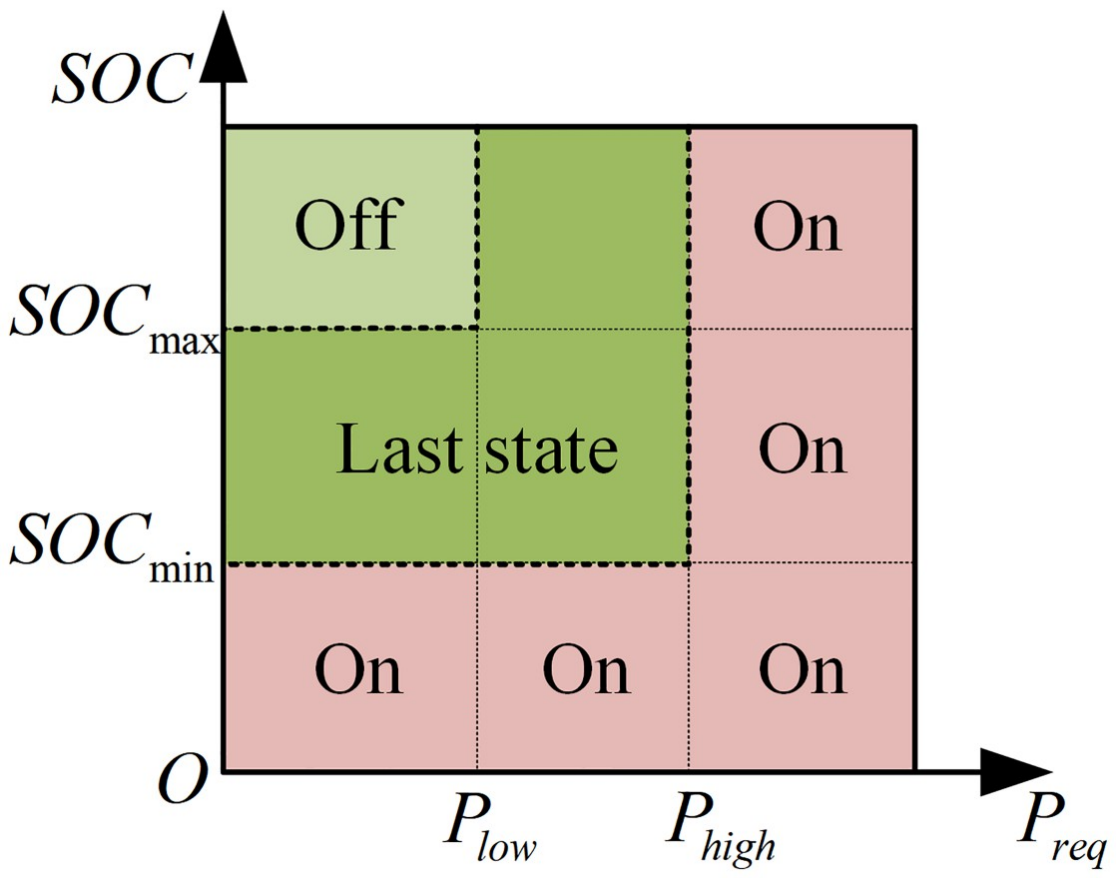
Power-following control logic.

PFCS is a rule-based control strategy, which can be categorized into two types: PFCS which tracks the demand power, and PFCS which tracks the SOC of the battery [22]. This paper adopts the PFCS that tracks demand power. When the tractor is under high-power load, the engine is activated. When the battery SOC exceeds the upper limit and the load is low, the engine is turned off. At all other times, the engine maintains its previous state. The control logic is illustrated in Fig 8.

**Fig 9:**
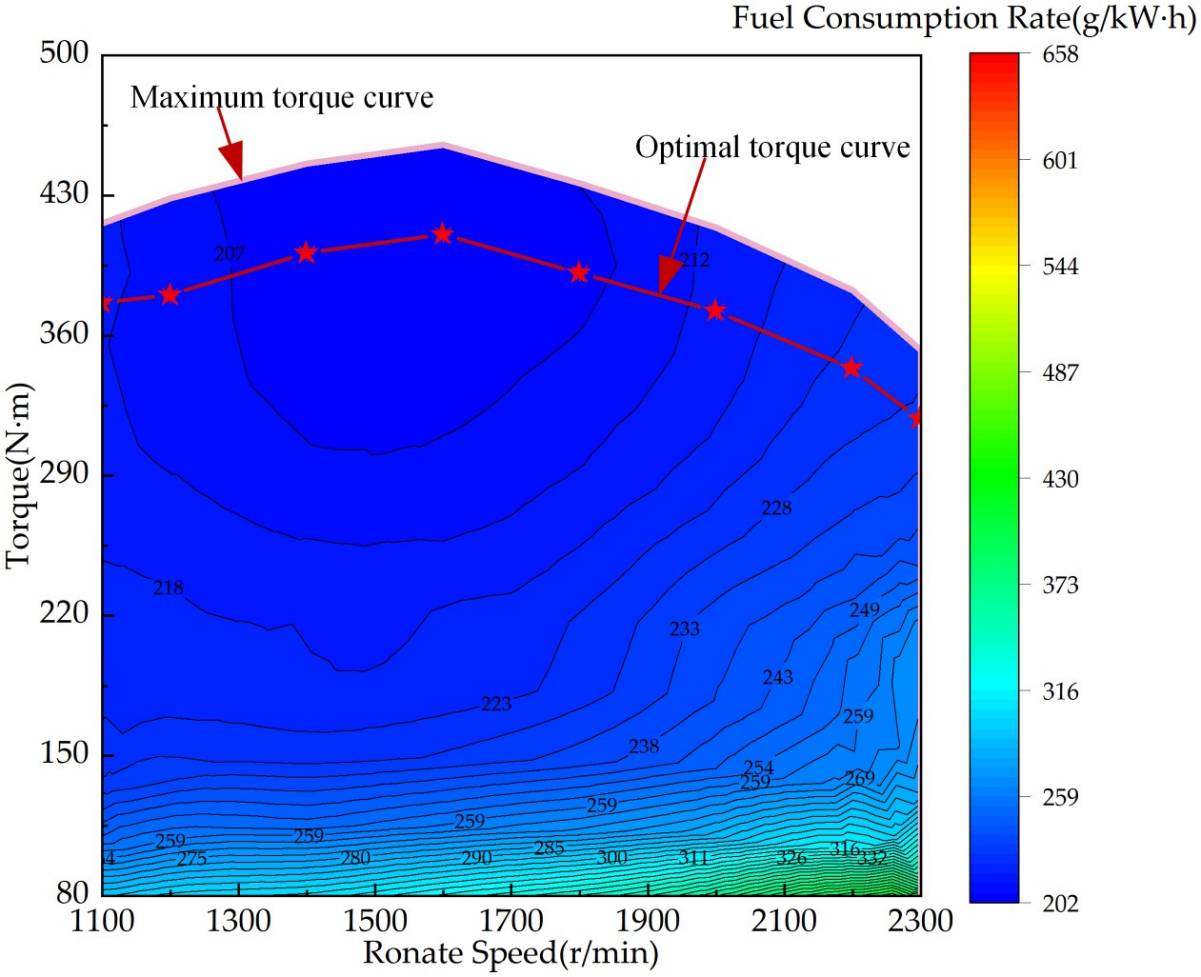
Engine universal characteristic map.

The PFCS developed in this study ensures that the engine follows the overall tractor power demand while operating along its optimal torque curve, as shown in Fig 9.

#### FCS

Traditional PFCS can lead to frequent engine start-stop cycles, which undermines the advantages of hybrid tractors and does not significantly improve engine fuel economy. Therefore, this paper enhances the PFCS by incorporating fuzzy control. In this approach, the power battery SOC and the overall tractor torque demand are used as fuzzy control input variables, while engine torque is the fuzzy control output variable. This adjustment aims to maximize the overall efficiency of the transmission system and achieve efficient energy flow throughout the tractor.

Based on the actual operating conditions of the hybrid tractor, a dual-input single-output fuzzy logic controller is configured with the power battery SOC and demand torque *T*_*req*_ as inputs, and engine torque *T*_*e*_ as the output. The fuzzy inference process is employed to achieve reasonable control of the overall energy flow in the tractor.

The motor demand torque *T*_*m*_ can be determined as:

$$T_{e}=T_{req}-T_{e}$$ (12)

**Fig 10:**
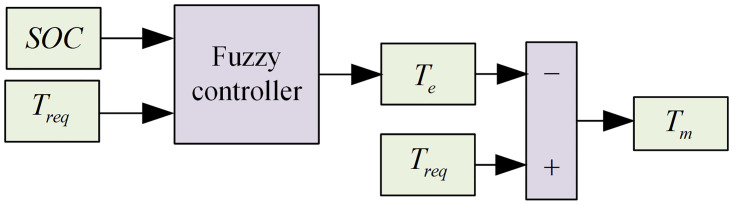
Fuzzy controller structure diagram.

The structure of the fuzzy controller is shown in Fig 10.

**Fig 11:**
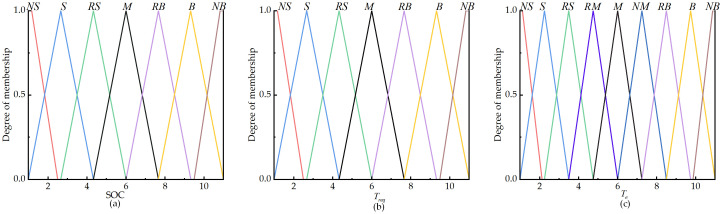
Membership function graph. (a) Membership function graph of SOC. (b) Membership function graph of *T*_*req*_. (c) Membership function graph of *T*_*e*_.

The current ranges for the power system’s battery SOC and demand torque, as well as engine torque, are set as [0.3, 0.8], $\left[0,T_{req_{\text{max}}}\right]$, and $\left[0,T_{e_{\text{max}}}\right]$, respectively. Based on the tractor’s actual operating conditions and considering factors such as power source lag, the linguistic variables are categorized into fuzzy subsets. The power battery SOC and demand torque *T*_*req*_ are divided into 7 fuzzy subsets: {NS, S, RS, M, RB, B, NB}, with the domain quantified as [1, 11]. The engine torque *T*_*e*_ is divided into 9 subsets: {NS, S, RS, RM, M, NM, RB, B, NB}, with the domain quantified as [1, 11]. Here, NS (very small), S (small), RS (relatively small), RM (medium-small), M (medium), NM (medium-large), RB (relatively large), B (large), and NB (very large). The model uses the Mamdani algorithm with triangular and trapezoidal membership functions, as illustrated in Fig 11.

Based on the operational conditions of a parallel hybrid tractor, the following fuzzy rules are established:

When the tractor’s torque demand is low, such as during startup, the tractor is driven solely by the electric motor.

When the SOC of the power battery is high, the electric motor alone drives the tractor to avoid operation in low-efficiency regions. If the maximum torque of the electric motor is less than the required torque, both the electric motor and the engine will jointly drive the tractor.

When the SOC of the power battery is low, the engine serves as the primary power source, while simultaneously charging the power battery to ensure normal tractor operation.

**Fig 12:**
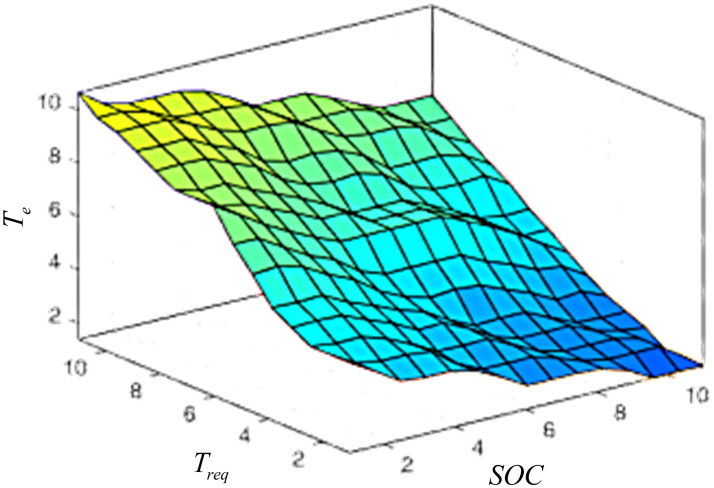
The fuzzy rules output surface of *T*_*e*_.

Using the above rules as a baseline, the fuzzy control rules are formulated in the "IF A and B, THEN C" conditional statement format, as shown in Table 2. The fuzzy controller is designed using the Fuzzy Logic Toolbox on Matlab, with the fuzzy rules implemented, and the output surface of the fuzzy rules is illustrated in Fig 12.

**Table 2:** Fuzzy control rules.

*T*_*e*_	SOC							
		NS	S	RS	M	RB	B	NB
***T***_***req***_	**NS**	RM	RS	RS	S	S	RS	NS
	**S**	RM	RM	RS	RS	S	S	NS
	**RS**	M	M	RM	RS	RS	S	S
	**M**	RB	NM	M	M	M	RM	RS
	**RB**	RB	RB	NM	M	M	RM	RM
	**B**	B	B	RB	RB	NM	NM	M
	**NB**	NB	B	B	RB	RB	NM	NM

### AIPSOFCS

Since fuzzy rules are derived from empirical reasoning, ensuring optimal control results cannot be guaranteed. Therefore, to enhance the fuel economy of tractors, the AIPSO is employed to optimize the fuzzy rules, aiming to achieve better control performance and effectively reduce the equivalent fuel consumption of tractors.

#### AIPSO

AIPSO is an improvement and extension of the traditional Particle Swarm Optimization (PSO) and Immune Algorithms (IA), which effectively enhances particle convergence speed and global search capability while increasing robustness and adaptability to better suit the problem-solving process in various scenarios [23, 24]. Although AIPSO has been widely applied in fields such as engineering optimization, signal processing, and path planning, it has not yet been utilized in the research of energy optimization control strategies for parallel hybrid tractors [25, 26].

In PSO, the updated formula for particle velocity is given by:

$$v_{i,d}^{k+1}=\omega^{k}v_{i,d}^{k}+c_{1}r_{1}\left(x_{p,d}^{k}-x_{i,d}^{k}\right)+c_{2}r_{2}\left(x_{g,d}^{k}-x_{i,d}^{k}\right)$$ (13)

The updated formula for particle position is given by:

$$x_{i,d}^{k+1}=x_{i,d}^{k}+v_{i,d}^{k+1}$$ (14)

Where *v* represents the velocity of the particle; *x* represents the position of the particle; *i* represents the encoding of particle; *k* represents the iterations of particle; *d* represents the dimensionality of particle; *x*_*d*_ represents the position of the individual best solution for particle; *x*_*g*_ represents the position of the global best solution; *c*_1_ represents the individual learning factor; *c*_2_ represents the individual learning factor; *r*_1_ and *r*_2_ are random numbers within the range [0,1], which effectively increase the randomness of the search process; *ω* represents the inertia weight.

**Fig 13:**
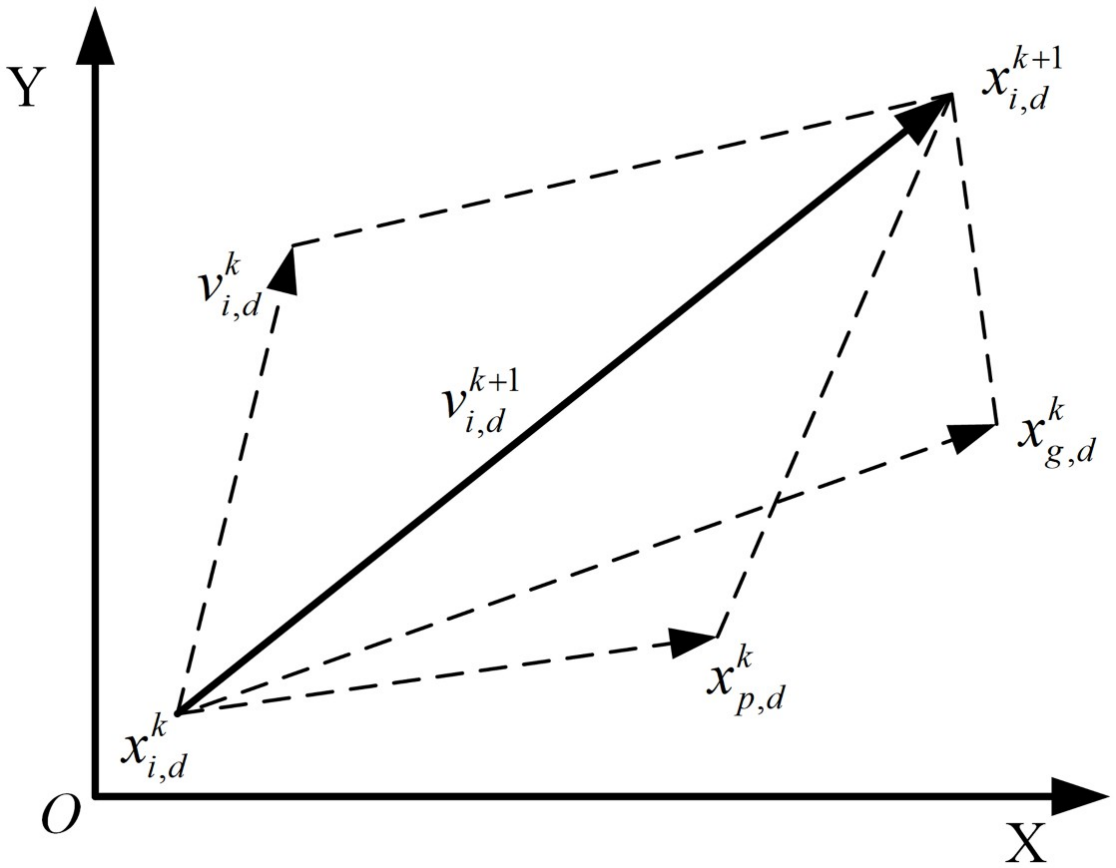
Particle movement diagram.

The illustration of particle movement during the iteration process is shown in Fig 13.

The PSO algorithm currently faces two major challenges: convergence speed and local minimums, which impose significant limitations on its application. In contrast, the Adaptive Particle Swarm Optimization (APSO) algorithm utilizes a cosine-based variation in the average particle velocity for searching, which results in higher efficiency. In the later stages of the search process, particles maintain smaller values for extended periods, leading to a substantial improvement in precision [27].

As a crucial parameter in the iteration process, the inertia weight ω significantly impacts the ability of the PSO algorithm to find the optimal solution. A larger ω enhances the global search capability of particles, helping the algorithm to escape local optima, while a smaller ω improves the local search capability, leading the algorithm towards convergence [28, 29]. Therefore, designing an effective inertia weight adjustment mechanism is highly beneficial for the algorithm’s performance. Common methods for improving inertia weight include adaptive weighting, random weighting, and linear decreasing weighting [30–32]. This paper employs the linear decreasing weight method, where the inertia weight ω is dynamically adjusted according to the iteration count *k*, with the adjustment formula given by [33]:

$$w^{k}=w_{\text{max}}-k\frac{\left(w_{\text{max}}-w_{\text{min}}\right)}{K}$$ (15)

Where *ω*_max_ is the maximum value of the inertia weight, taken value as 0.9; *ω*_min_ is the minimum value of the inertia weight, taken value as 0.5; *K* is the minimum value of the inertia weight.

During the particle swarm search process, the focus is initially on individual self-recognition capability and later on social recognition capability. The individual learning factor *c*_1_ represents the particle’s self-learning ability, reflecting the extent of the particle’s personal experience. The social learning factor *c*_2_ represents the particle’s ability to learn from better-performing particles in the swarm, indicating the particle’s social recognition capability. Therefore, setting nonlinear variations of *c*_1_ and *c*_2_ can effectively enhance the algorithm’s global search and local exploration abilities. The formula for the variation of the learning factors is [34]:

$$\left\{\begin{array}{c} c_{1}=\alpha \times \text{sin}((1-\frac{k}{K})\times \frac{\pi }{2})+\beta \\ c_{2}=\alpha \times \text{cos}((1-\frac{k}{K})\times \frac{\pi }{2})+\beta \end{array}\right.$$ (16)

In the equation, *α* = 2; *β* = 0.5. In the initial state, *c*_1_ is 2.5, *c*_2_ is 0.5.

IA originates from the immune system of natural organisms. By simulating the process through which the immune system of biological entities identifies antigens and produces antibodies, IA seeks to optimize problems. This algorithm is a heuristic stochastic search method that combines determinism with randomness and exploration with exploitation [35]. PSO algorithms often become trapped in local extrema during the optimization process and fail to fully converge to a global optimum. As the optimization progresses to later stages, the efficiency decreases, and a single PSO algorithm may not achieve high convergence accuracy. To address this, an AIPSO algorithm is proposed. By incorporating immune algorithms, AIPSO effectively improves the premature convergence characteristic of PSO. It retains high-quality particles during the iteration process while ensuring particle diversity to avoid local optima and achieve global optimum.

In the AIPSO algorithm, antigens represent the optimal solution to the objective function, antibodies represent particles, and the affinity between antibodies and antigens indicates the degree of match between the current solution and the optimal solution. The concentration of antibodies represents the level of population diversity. The particle selection is based on the antibody concentration adjustment mechanism, which effectively enhances the diversity of the particle swarm and improves the algorithm’s optimization capability. Immune selection operations refer to the process where antibodies with a comprehensive selection probability greater than a predetermined immune replacement probability are replaced. This operation effectively improves the convergence speed of the algorithm [36].

Affinity refers to the fitness of the antibodies concerning the antigen and the concentration among the antibodies, as represented by the following formula:

$$p(x_{i})=\alpha f(x_{i})+\left(1-\alpha \right)d(x_{i})$$ (17)

Where *p*(*x*_*i*_) represents the affinity value of the *i*_*th*_ particle, *f*(*x*_*i*_) denotes the fitness value of the *i*_*th*_ particle, *d*(*x*_*i*_) is the concentration value of the *i*_*th*_ particle, and *α* is the weight coefficient.

#### Parameters to be optimized by AIPSO

**Fig 14:**
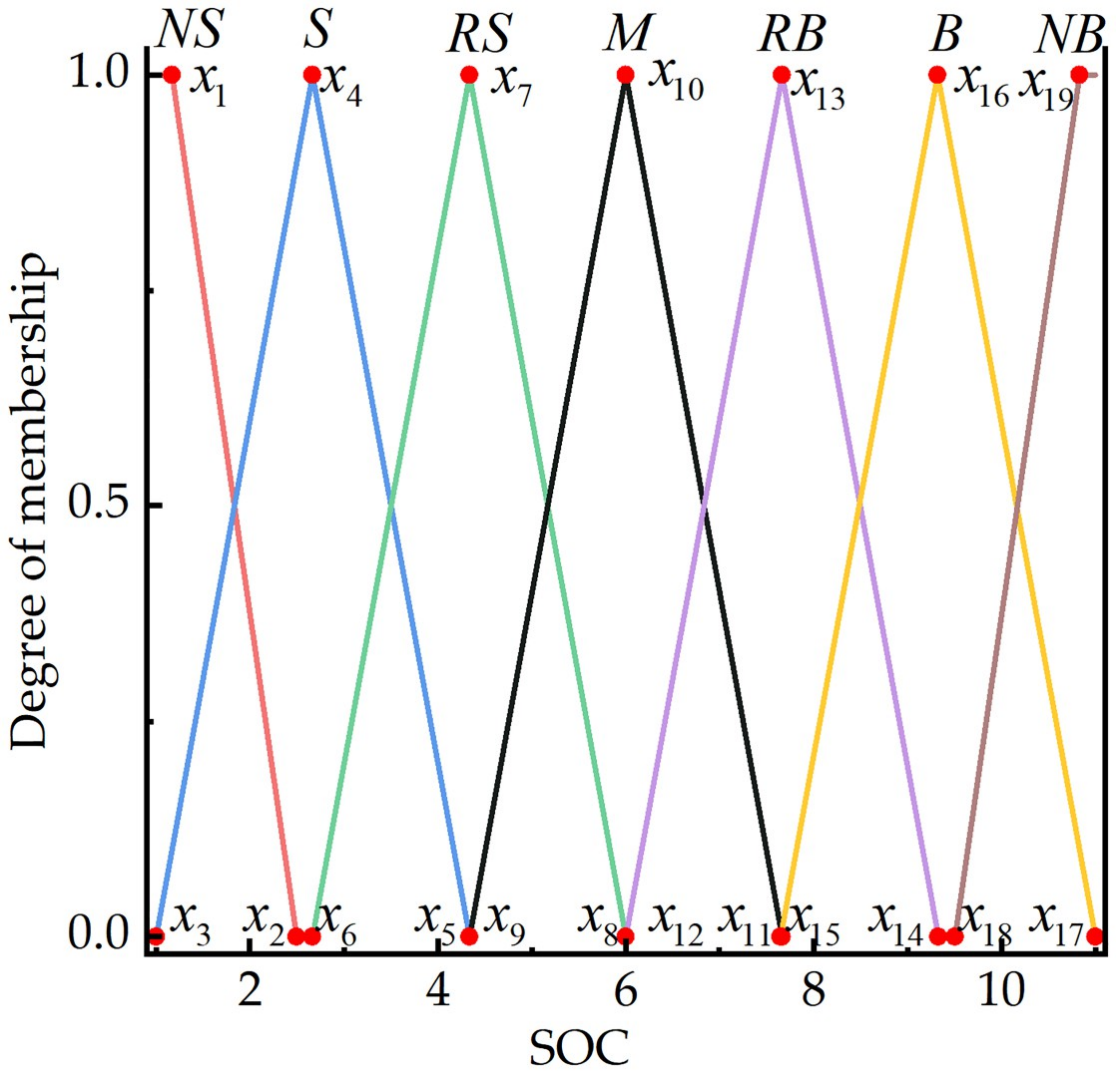
Parameters to be optimized for the SOC membership function.

In a fuzzy controller, each fuzzy subset corresponds to a membership function. For triangular membership functions, there are three parameters to be optimized. For the trapezoidal membership function at both ends, there are only two parameters to be optimized. Taking the SOC of a power battery as an example, the required membership function parameters for optimization are illustrated in Fig 14.

The fuzzy controller discussed in this paper has two inputs and one output, corresponding to a total of 23 fuzzy subsets. With the coordinates of each fuzzy subset serving as optimization variables, there are 63 parameters to be optimized. The optimization ranges for these parameters are shown in Table 3.

**Table 3:** Membership function parameter optimization range.

Parameters to be optimized	Optimization Range
*x*_1_, *x*_3_, *x*_20_, *x*_22_, *x*_39_, *x*_41_	[1 2]
*x*_2_, *x*_4_, *x*_6_, *x*_21_, *x*_23_, *x*_25_, *x*_40_, *x*_42_, *x*_44_	[2 3]
*x*_45_, *x*_47_	[3 4]
*x*_5_, *x*_7_, *x*_9_, *x*_24_, *x*_26_, *x*_28_, *x*_43_, *x*_46_, *x*_48_, *x*_50_	[4 5]
*x*_8_, *x*_10_, *x*_12_, *x*_27_, *x*_29_, *x*_31_, *x*_49_, *x*_51_, *x*_53_,	[5.5 6.5]
*x*_11_, *x*_13_, *x*_15_, *x*_30_, *x*_32_, *x*_34_, *x*_52_, *x*_54_, *x*_56_	[7 8]
*x*_55_, *x*_57_, *x*_59_	[8 9]
*x*_14_, *x*_16_, *x*_18_, *x*_33_, *x*_35_, *x*_37_, *x*_38_, *x*_60_	[9 10]
*x*_62_	[9.5 10.5]
*x*_17_, *x*_19_, *x*_36_, *x*_38_, *x*_61_	[10 11]
*x*_63_	[10.5 11]

**Fig 15:**
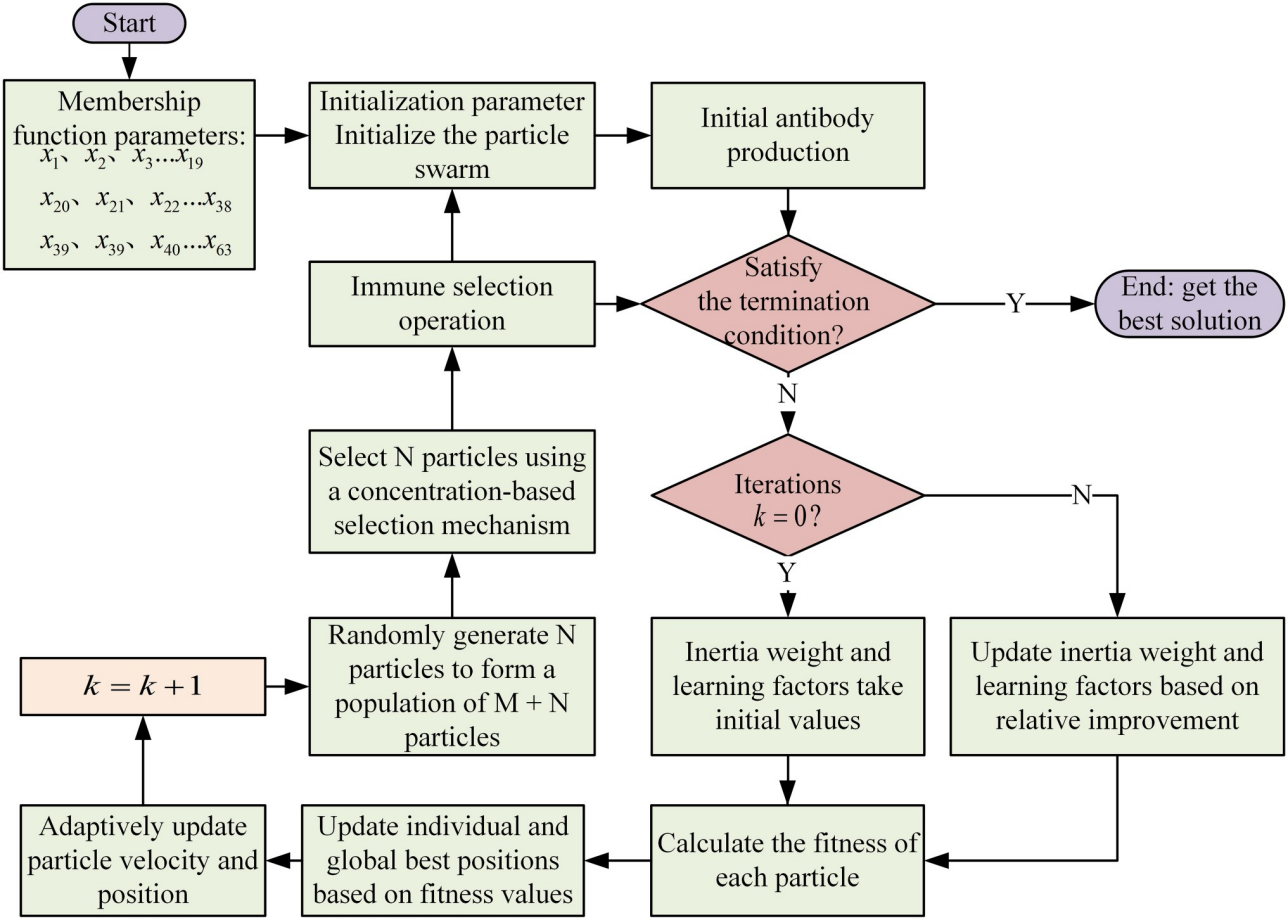
AIPSO algorithm flowchart.

The objective of this paper is to minimize the equivalent fuel consumption of the tractor. The flowchart of the AIPSO algorithm is shown in Fig 15.

The parallel hybrid tractor discussed in this paper has two power sources. The energy consumption of the tractor includes both fuel consumption and electrical energy consumption. Therefore, an equivalent fuel consumption calculation model based on the heating value method is established as an observation metric during the AIPSO iteration process.

$$V_{fueleq}=V_{fuelen}+V_{fuelba}$$ (18)

$$V_{fuelba}=\frac{Q}{k_{b}}$$ (19)

$$Q=3.6\times 10^{6}\times (SOC_{end}-SOC_{start}\text{)}\times W_{ba}$$ (20)

$$k_{b}=\rho \eta_{d}$$ (21)

Where *V*_*fueleq*_ represents the equivalent fuel consumption, L; *V*_*fuelen*_ denotes the engine fuel consumption, L; *V*_*fuelba*_ is the equivalent fuel consumption of the power battery, L; *Q* is the electrical energy consumption of the power battery, J; *k*_*b*_ is the energy conversion coefficient based on the heating value method; *SOC*_*end*_ is the SOC of the power battery at the end of the operation; *SOC*_*start*_ is the SOC of the power battery at the start of the operation; *W*_*ba*_ is the power of the battery, kW·h; *ρ* is the density of diesel fuel, 0.83 kg/L; *η*_*d*_ is the heating value of diesel fuel per unit quantity, J/kg.

To ensure the operational safety of the tractor’s power system, the SOC of the power battery, as well as the engine speed and torque, need to be restricted within certain ranges. Therefore, the constraint ranges for the SOC, discharge power of the power battery, and optimized variable parameters are set as shown in Eq (22).


$$\left\{\begin{array}{c} \begin{array}{c} \begin{array}{c} SOC_{\text{min}}<SOC<SOC_{\text{max}}\\ P_{batt,\text{min}}<P_{batt}<P_{batt,\text{max}} \end{array}\\ T_{eng,\text{min}}<T_{eng}<T_{eng,\text{max}} \end{array}\\ w_{eng,\text{min}}<w_{eng}<w_{eng,\text{max}} \end{array}\right.$$ (22)

**Fig 16:**
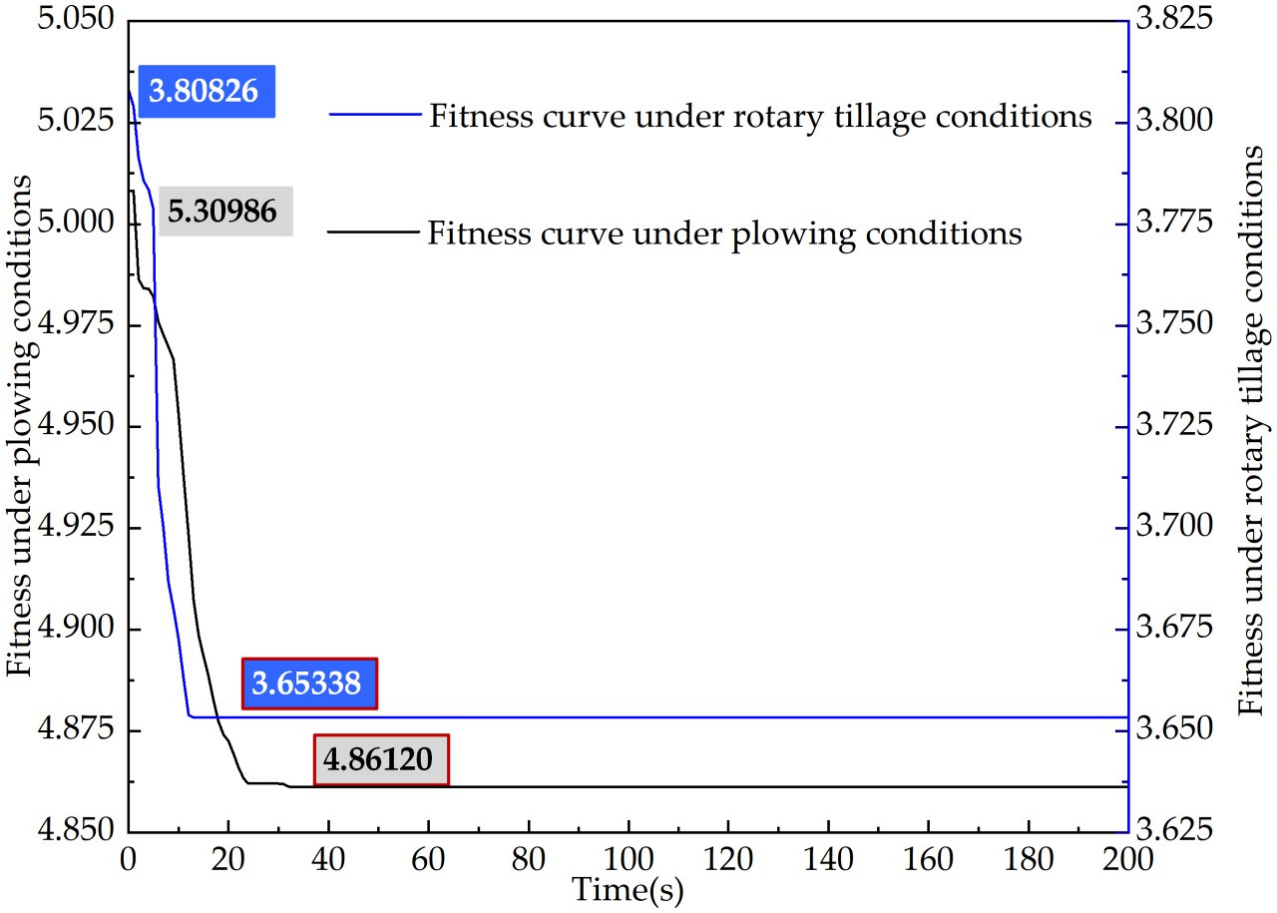
Curve of AIPSO iteration.

**Fig 17:**
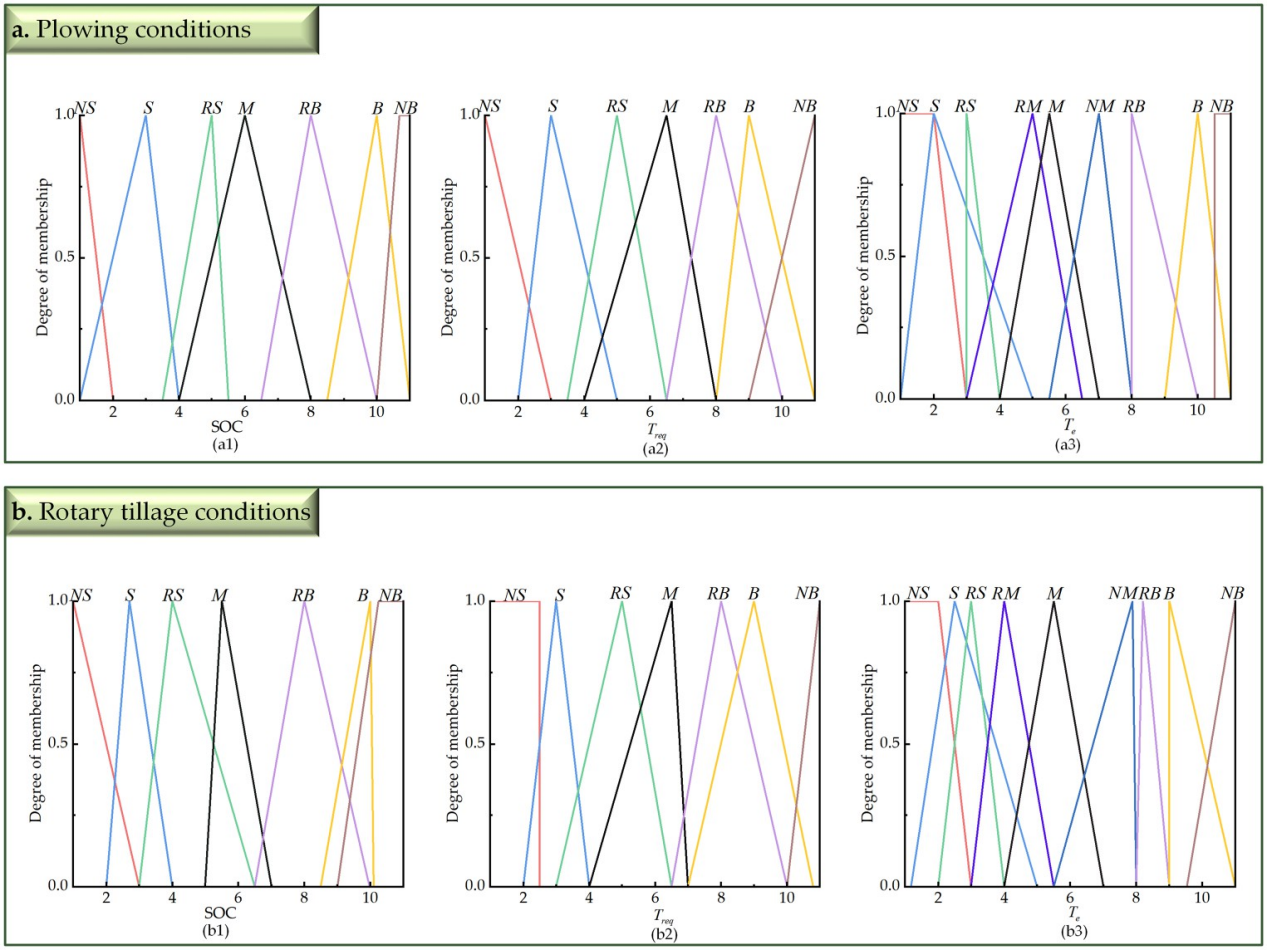
Optimized membership function graph. (a) Membership function graph after optimization under plowing conditions. (a1) Optimized membership function graph of SOC under plowing conditions. (a2) Optimized membership function graph of *T*_*req*_ under plowing conditions. (a3) Optimized membership function graph of *T*_*e*_ under plowing conditions. (b) Membership function graph after optimization under rotary tillage conditions. (b1) Optimized membership function graph of SOC under rotary tillage conditions. (b2) Optimized membership function graph of *T*_*req*_ under rotary tillage conditions. (b3) Optimized membership function graph of *T*_*e*_ under rotary tillage conditions.

In the algorithm optimization process of this paper, the horizontal coordinates of the membership function points are selected as optimization variables to construct the particle swarm. The initial number of particles in the swarm is set to 20; the maximum number of iterations is 200; the dimensionality is 63; the inertia weight is selected according to Eq (15); and the learning factors are selected according to Eq (16). The iteration curve is shown in Fig 16, and the membership functions of the optimized fuzzy controller based on the plowing and rotary tillage conditions of the tractor are shown in Fig 17.

## Simulation results of plowing conditions and rotary tillage conditions

The primary work of tractors involves field operations such as plowing, rotary tillage, and transportation while equipped with agricultural machinery. Based on the main cultivation types of tractors in China and the farming habits of local farmers, it is found that plowing and rotary tillage are the predominant operational modes for tractors. Therefore, this study employs Matlab/Simulink simulation software to establish a control strategy model, selecting plowing and rotary tillage conditions for simulation analysis. A comparative analysis of the advantages and disadvantages of the aforementioned control strategies is conducted, along with HIL simulation testing of the controller to validate the effectiveness of these control strategies.

### Operating condition settings

To verify the feasibility and effectiveness of the energy optimization control strategy designed for the parallel hybrid tractor, performance simulations of three control strategies are conducted under both plowing and rotary tillage conditions.

#### Plowing conditions

**Fig 18:**
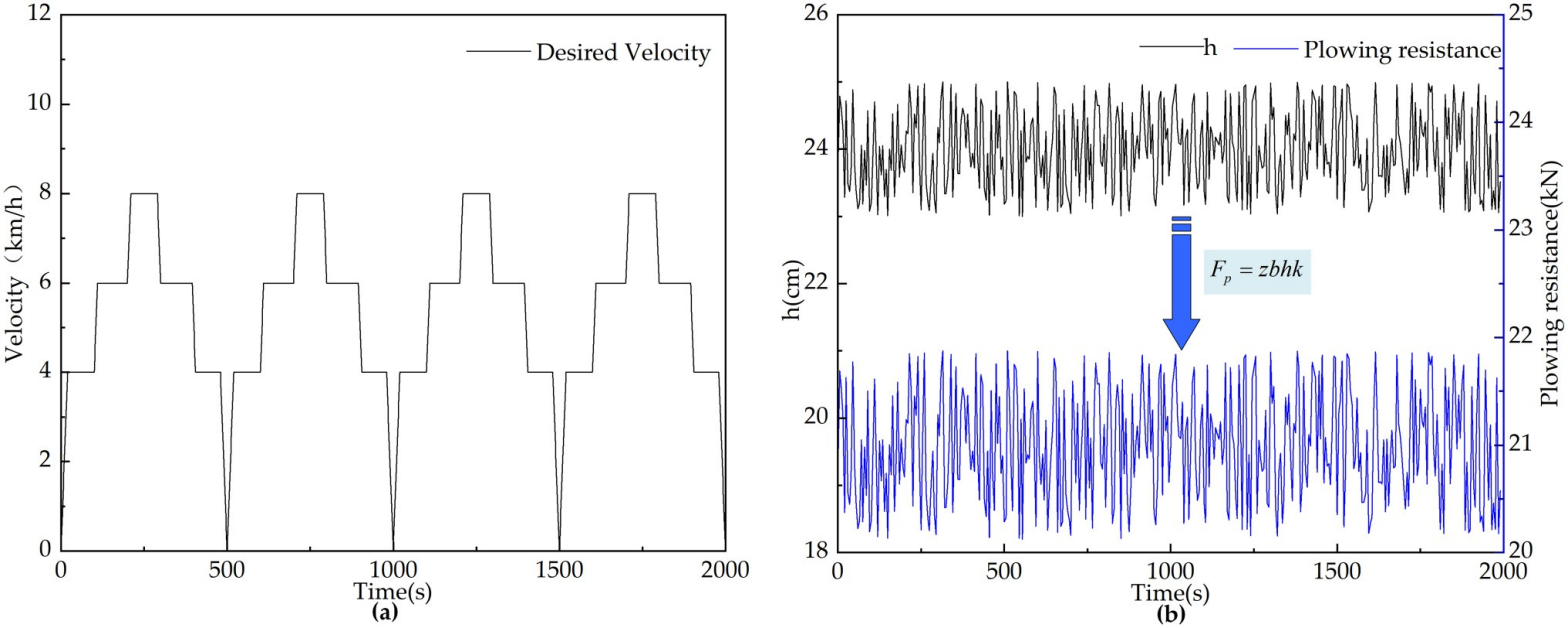
Changes of relevant parameters of tractor plowing conditions. (a) Desired velocity. (b) Plowing depth and plowing resistance.

To verify the feasibility and effectiveness of the energy optimization control strategies designed for the parallel hybrid tractor, performance simulations are conducted for three different control strategies under plowing operation conditions. To test the effectiveness of the control strategies for various mode switches, the plowing conditions are set to include uniform acceleration, uniform speed, and uniform deceleration, with the uniform plowing operation speed controlled between 4–7 km/h. Each operational cycle lasts 500 seconds, and the simulations are run for 4 cycles, resulting in a total continuous operating time of 2000 seconds for the hybrid tractor. The speed variations during plowing are shown in Fig 18(a). During plowing, the tractor load is dynamically changing. Therefore, in the simulation experiments, to closely approximate the real working conditions of the tractor, a variable random load is simulated. The plowing depth is used as a variable parameter, and random numbers within a range of 24 ± 1 cm are generated in Matlab/Simulink software. Based on this, the variable plowing resistance of the tractor is simulated, as shown in Fig 18(b).

#### Rotary tillage conditions

**Fig 19:**
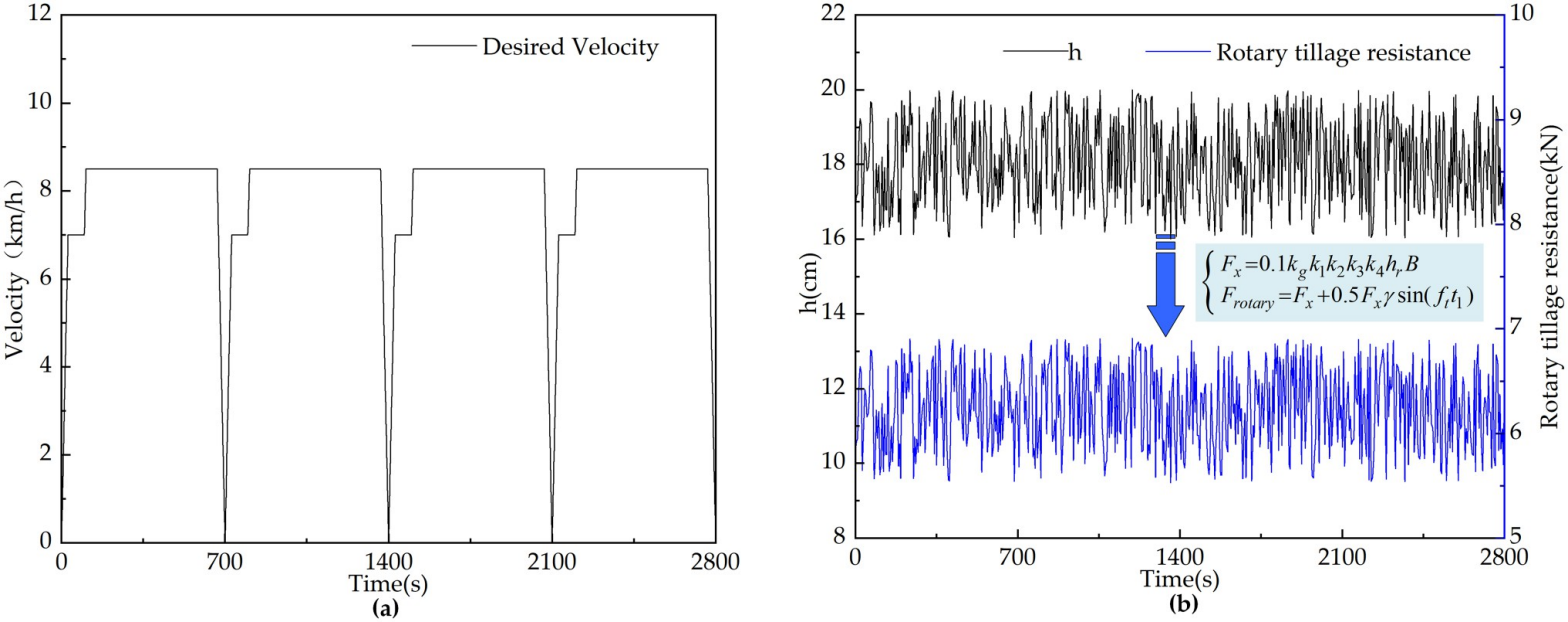
Changes of relevant parameters of tractor rotary tillage conditions. (a) Desired velocity. (b) Rotary tillage depth and rotary tillage resistance.

The tractor’s rotary tillage operation speed curve is established according to actual working speeds. As shown in Fig 19(a), the period from 0 to 665 seconds represents the outbound operation phase, consisting of three conditions: constant acceleration, constant speed, and constant deceleration. The interval from 665 to 700 seconds covers the tractor’s turning phase, and from 700 to 1400 seconds represents the return operation phase, with the same conditions as the outbound phase. During rotary tillage, the tractor load varies dynamically.

To closely replicate real working scenarios in the simulation experiments, a variable random load is simulated. The tilling depth is treated as a variable parameter, and random numbers of 15 ± 1 cm are generated in Matlab/Simulink to represent the depth. Based on references [37, 38], the rotary tillage resistance is simulated, as illustrated in Fig 19(b).

### Matlab/Simulink simulation results

Simulation analysis is a crucial method for verifying the rationality of the proposed control strategies. In this study, Matlab/Simulink simulation software is used to establish the control strategy model. Simulation experiments are conducted for both plowing and rotary tillage conditions to compare and analyze the advantages and disadvantages of the proposed control strategies.

#### Plowing conditions

**Fig 20:**
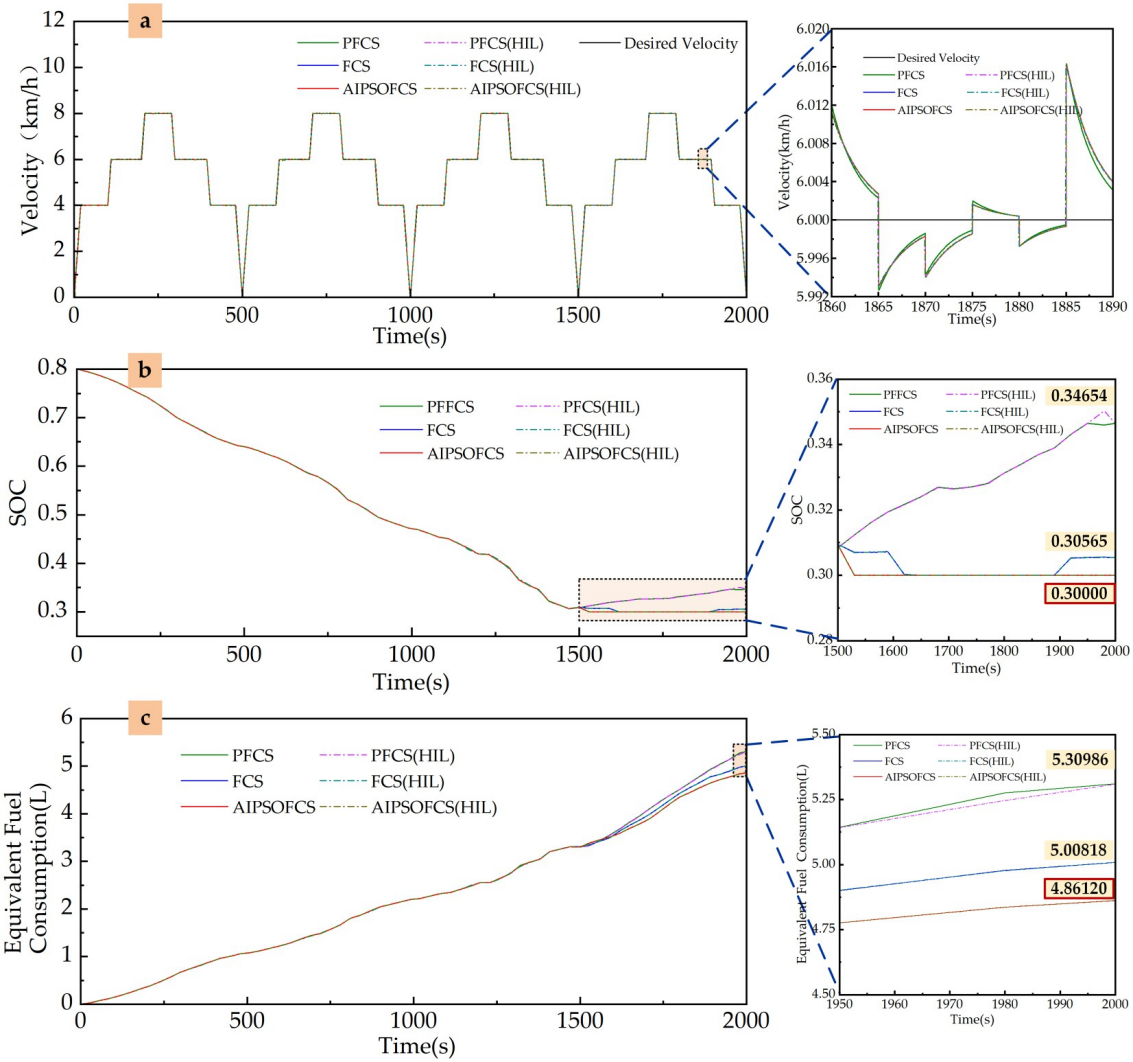
Simulation results of three energy optimization control strategies under plowing conditions using Matlab/Simulink and HIL. (a) Velocity. (b) SOC. (c) Equivalent fuel consumption.

For the plowing conditions of the tractor shown in Fig 18, simulations are conducted for three control strategies: PFCS, FCS, and AIPSOFCS. The simulation results are compared as follows: the desired velocity versus the actual velocity of the three control strategies is shown in Fig 20(a); the variation in the SOC of the power battery is illustrated in Fig 20(b); and the changes in equivalent fuel consumption are depicted in Fig 20(c). The related simulation results for the three control strategies are summarized in Table 4.

**Table 4:** Correlation results of three energy optimization control strategies under plowing conditions.

Control Strategy	Final SOC Value	Change in SOC	Fuel Consumption(L)	Equivalent Fuel Consumption(L)
PFCS	0.34654	0.45346	2.27018	5.30980
FCS	0.30545	0.49465	1.69310	5.00818
AIPSOFCS	0.30000	0.50000	1.50961	4.86120

The comparison of desired velocity and actual velocity is shown in Fig 20(a), demonstrating that the actual speed effectively tracks the target speed.

The variation in the SOC of the power battery is illustrated in Fig 20(b) and Table 4. Under the PFCS, the final SOC value is 0.34654; under the FCS, the final SOC value is 0.30545; and under the AIPSOFCS, the final SOC value is 0.30000.

The changes in equivalent fuel consumption are presented in Fig 20(c) and Table 4. The equivalent fuel consumption is highest under the PFCS control strategy and lowest under the AIPSOFCS control strategy.

#### Rotary tillage conditions

**Fig 21:**
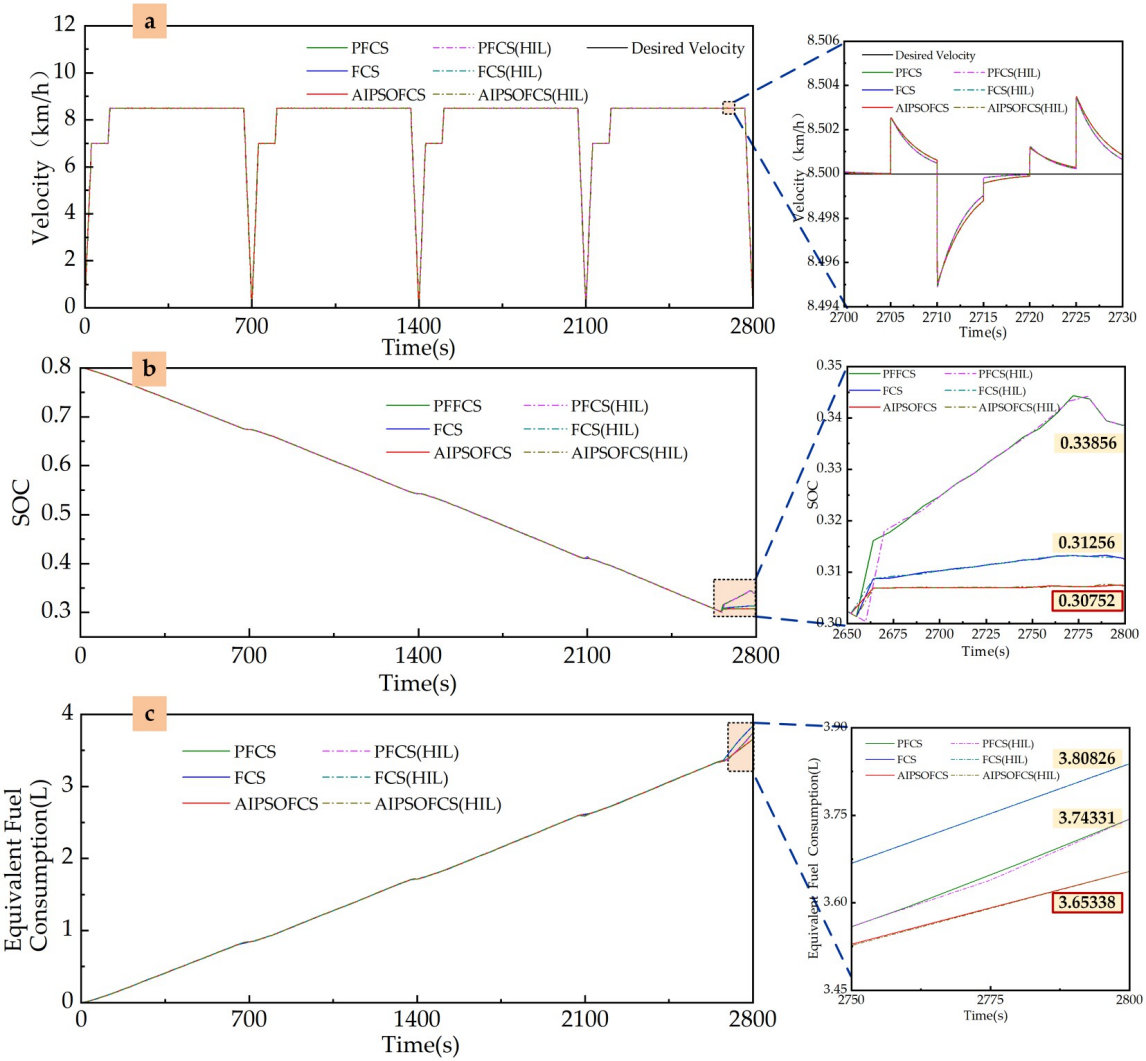
Simulation results of three energy optimization control strategies under rotary tillage conditions using Matlab/Simulink and HIL. (a) Velocity. (b) SOC. (c) Equivalent fuel consumption.

For the rotary tillage condition illustrated in Fig 19, simulation analyses were performed for three control strategies: PFCS, FCS, and AIPSOFCS. The simulation results include a comparison between the target vehicle speed and the simulated speed, as shown in Fig 21(a). The variation in the SOC of the power battery is presented in Fig 21(b), while the change in equivalent fuel consumption is depicted in Fig 21(c). A summary of the simulation results for the three control strategies is provided in Table 5.

**Table 5:** Correlation results of three energy optimization control strategies under rotary tillage conditions.

Control Strategy	Final SOC Value	Change in SOC	Fuel Consumption(L)	Equivalent Fuel Consumption(L)
PFCS	0.33856	0.46144	0.65025	3.74331
FCS	0.31256	0.48744	0.54102	3.80826
AIPSOFCS	0.30752	0.49248	0.35081	3.65338

The comparison between the target vehicle speed and the simulated speed is shown in Fig 21(a), demonstrating that all three control strategies effectively track the target speed.

The SOC variation of the power battery is presented in Fig 21(b) and summarized in Table 5. The final SOC values are 0.33856 under the PFCS strategy, 0.31256 under the FCS strategy, and 0.30752 under the AIPSOFCS strategy.

The equivalent fuel consumption is illustrated in Fig 21(c) and detailed in Table 5. Among the three strategies, the FCS strategy results in the highest equivalent fuel consumption, while the AIPSOFCS strategy achieves the lowest.

### HIL testing results

**Fig 22:**
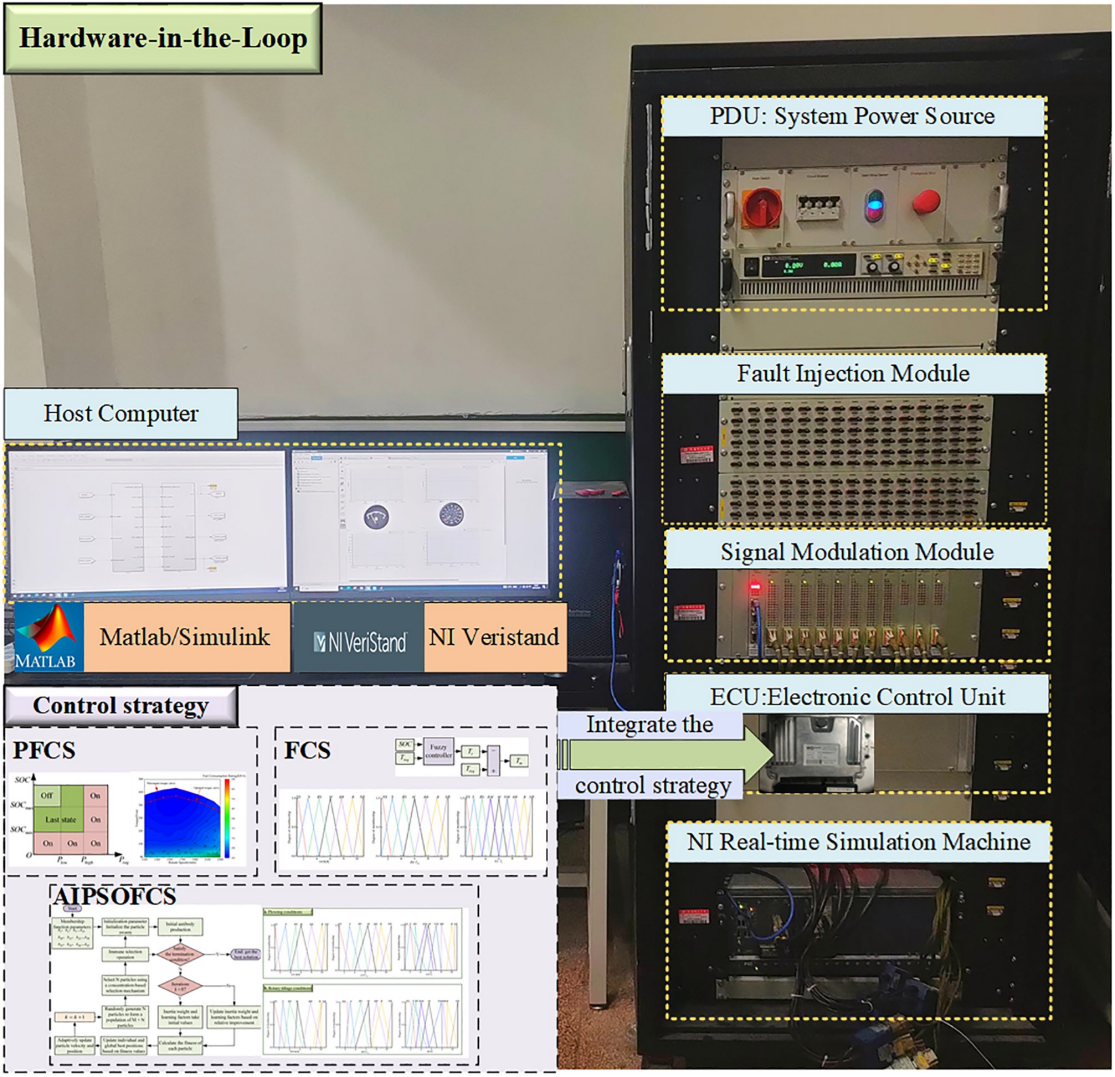
Platform of HIL system.

To further validate the control effectiveness of the three strategies in an offline simulation environment, this paper builds a HIL testing platform based on offline simulations using NI Veristand software in conjunction with an NI real-time simulation machine. By importing the power system model, a rapid HIL testing platform is constructed for simulation experiments. The results from both simulations are compared. The HIL testing platform is illustrated in Fig 22.

By setting up an HIL testing platform, the energy optimization control strategies proposed in this paper are downloaded to a real controller for validation in a real-time simulation environment. Control data is transmitted to the real-time simulation machine via NI boards, which then runs the power system model and outputs the processed data to the complete system. Additionally, this data can be fed back to the NI Veristand visualization interface for operator observation [39].

HIL simulation tests were conducted for the plowing and rotary tillage conditions shown in Figs 18 and 19, using three control strategies: PFCS, FCS, and AIPSOFCS. The results of vehicle speed variation, SOC change, and equivalent fuel consumption from the HIL tests closely align with the Matlab/Simulink simulation results. The trends of the relevant parameters are illustrated in Figs 20 and 21.

## Discussion

Analysis and discussion of Matlab/Simulink Simulation results and HIL testing results under plowing conditions:

1. Velocity: Under all three control strategies, the actual vehicle speed in both plowing and rotary tillage conditions effectively track the target speed, meeting the overall power requirements of the machine. This confirms the correctness of the model, the effectiveness of the control strategies, and the accuracy of the simulation environment.
2. SOC: In both plowing and rotary tillage conditions, the SOC of the power battery exhibits similar variation patterns across the three control strategies. The final SOC value is highest under the PFCS, while the AIPSOFCS maintains a stable SOC, indicating that AIPSOFCS has good battery management performance.
3. Equivalent fuel consumption: Among the three control strategies, AIPSOFCS performs best in reducing equivalent fuel consumption. In the plowing conditions, AIPSOFCS reduces equivalent fuel consumption by 8.45% and 2.93% compared to PFCS and FCS, respectively. In the rotary tillage conditions, AIPSOFCS achieves reductions of 2.40% and 4.07% compared to PFCS and FCS, respectively. These results in both working conditions demonstrate the significant energy-saving advantages of AIPSOFCS.

Although there are certain lag discrepancies between the HIL test results and the simulation results under both plowing conditions and rotary tillage conditions, the overall trends are consistent. This effectively validates the effectiveness of the proposed AIPSOFCS in real controllers and the reliability of this control strategy in practical applications.

The control strategy proposed in this study focuses on torque and employs the AIPSO algorithm to optimize the fuzzy control membership functions. Although it achieves a slight improvement in tractor performance compared to the other two control strategies, there remains room for further optimization. Currently, several advanced optimization algorithms, such as skill optimization algorithms, seasonal optimization algorithms, and energy valley optimizers, have been proposed by various experts and scholars, but they have yet to be applied in the automotive and tractor fields. In future research, incorporating these advanced algorithms may further enhance the energy optimization control strategy, aiming to improve overall fuel economy and work efficiency for better control outcomes. Additionally, during the study of tractor plowing and rotary tillage conditions, only simulated data were used due to constraints, and actual data testing as well as real vehicle trials were not conducted. In subsequent research, if the testing equipment allows, field data can be collected under various agricultural environments for simulation analysis. Furthermore, a parallel hybrid tractor compatible with the power system discussed in this study could be developed for real vehicle testing, effectively validating the effectiveness of AIPSOFCS.

## Conclusions

1. This paper focuses on the parallel hybrid tractor, establishing a complete tractor model in Matlab/Simulink. It proposes a method based on AIPSO to optimize the fuzzy control membership functions and compare it with PFCS and FCS.
2. Under plowing conditions and rotary tillage conditions, simulations are conducted for the PFCS, FCS, and AIPSOFCS control strategies. The results demonstrate that all three strategies can effectively control the tractor. Under the plowing conditions, the equivalent fuel consumption of AIPSOFCS is reduced by 8.45% compared to PFCS and by 2.93% compared to FCS. Under the rotary tillage conditions, AIPSOFCS achieves a reduction in equivalent fuel consumption of 2.40% compared to PFCS and 4.07% compared to FCS.
3. A HIL testing platform was established, with the three proposed energy control strategies downloaded to the real controller. The control effects were validated in a real-time simulation environment, fully accounting for uncertainties in the actual controller to ensure the feasibility and effectiveness of the control strategies.
4. The designed AIPSOFCS integrates multiple algorithms, effectively reducing the equivalent fuel consumption of the parallel hybrid tractor. This research provides essential theoretical references and technical support for modeling and energy optimization control of parallel hybrid tractor systems.
